# FGF23 ameliorates ischemia-reperfusion induced acute kidney injury via modulation of endothelial progenitor cells: targeting SDF-1/CXCR4 signaling

**DOI:** 10.1038/s41419-021-03693-w

**Published:** 2021-04-17

**Authors:** Huang-Ming Chang, Kang-Yung Peng, Chieh-Kai Chan, Chiao-Yin Sun, Ying-Ying Chen, Han-Mei Chang, Chun-Lin Huang, Pei-Chun Liu, Peng-Ying Chen, Kuo-Chuan Wang, Wei-Jie Wang, Chen-Chi Wu, Yu-Feng Lin, Tai-Shuan Lai, Tao-Min Huang, Guang-Huar Young, Shuei-Liong Lin, Marlies Ostermann, Tzong-Shinn Chu, Jeff S. Chueh, Vin-Cent Wu

**Affiliations:** 1 Department of Internal Medicine, National Taiwan University Hospital, Taipei, Taiwan; 2 The National Taiwan University Study Group on Acute Renal Failure, Taipei, Taiwan; 3 Department of Internal Medicine, National Taiwan University Hospital, Hsin-Chu Branch, Hsin Chu, Taiwan; 4 Division of Nephrology, Chang Gung Memorial Hospital, Keelung, Taiwan; 5 Division of Nephrology, Department of Internal Medicine, MacKay Memorial Hospital, Taipei, Taiwan; 6 Department of Surgery, National Taiwan University Hospital, Taipei, Taiwan; 7 Department of Internal Medicine, Tao-Yuan General Hospital, Tao-Yuan County, Taiwan; 8 Department of Otolaryngology, National Taiwan University Hospital, Taipei, Taiwan; 9 Department of Critical Care and Nephrology, King’s College London, Guy’s and St Thomas Hospital, London, SE1 7EH UK; 10 Cleveland Clinic Lerner College of Medicine and Glickman Urological and Kidney Institute, Cleveland Clinic, Cleveland, OH USA

**Keywords:** Extracellular signalling molecules, DNA methylation, Acute kidney injury, Experimental models of disease

## Abstract

The levels of fibroblast growth factor 23 (FGF23) rapidly increases after acute kidney injury (AKI). However, the role of FGF23 in AKI is still unclear. Here, we observe that pretreatment with FGF23 protein into ischemia-reperfusion induced AKI mice ameliorates kidney injury by promoting renal tubular regeneration, proliferation, vascular repair, and attenuating tubular damage. In vitro assays demonstrate that SDF-1 induces upregulation of its receptor CXCR4 in endothelial progenitor cells (EPCs) via a non-canonical NF-κB signaling pathway. FGF23 crosstalks with the SDF-1/CXCR4 signaling and abrogates SDF-1-induced EPC senescence and migration, but not angiogenesis, in a Klotho-independent manner. The downregulated pro-angiogenic IL-6, IL-8, and VEGF-A expressions after SDF-1 infusion are rescued after adding FGF23. Diminished therapeutic ability of SDF-1-treated EPCs is counteracted by FGF23 in a SCID mouse in vivo AKI model. Together, these data highlight a revolutionary and important role that FGF23 plays in the nephroprotection of IR-AKI.

## Introduction

There is growing evidence indicating that fibroblast growth factor 23 (FGF23) levels are greatly elevated in mice and patients with acute kidney injury (AKI)^1–10^. Several clinical studies show that FGF23 levels can predict the development of severe AKI at its early stage^3–5,9,10^. Furthermore, increased circulating FGF23 levels are independently associated with adverse outcomes, such as requirement for renal replacement therapy, and mortality^2,4,6–8,10^. Although all of these studies suggested that FGF23 might be a direct toxic factor in AKI. To date, however, it has remained unclear whether FGF23 is a mere biomarker or is directly implicated in the pathogenesis of AKI, because there is no experimental evidence for that.

AKI, an abrupt deterioration of kidney function, is most commonly caused by renal ischemia^11^. Endothelial cell injury is central in the pathogenesis of ischemic and nephrotoxic AKI^12^. Following renal ischemia, vascular endothelial cell injury occurs and it further leads to tubular epithelial injury and inflammation^13^. Endothelial progenitor cells (EPCs) are a group of endothelial cell precursors with certain specific surface antigens and have been identified in bone marrow, umbilical cord blood, and peripheral blood circulation^14^. EPCs participate in postnatal angiogenesis^15,16^, and could attenuate ischemic AKI by increasing vascularization, as well as decreasing apoptosis, inflammation, and fibrosis^17^. However, whether FGF23 is linked to EPC-mediated renal repair is unknown.

SDF-1 (stromal cell-derived factor-1), also known as CXCL12 (C-X-C chemokine ligand 12), is a small pro-inflammatory chemoattractant cytokine for EPCs. SDF-1 binds to its receptor CXCR4 (C-X-C chemokine receptor type 4) and results in a variety of responses such as chemotaxis, cell survival, and proliferation^18^. Accumulating evidence shows that SDF-1 is upregulated in AKI^19^. Higher SDF-1 levels play a significant role in tissue repair by promoting the migration of EPCs to facilitate vascular repair^20,21^.

In this study, we show that FGF23 ameliorates ischemia-reperfusion induced AKI (IR-AKI) via modulation of SDF-1/CXCR4 signaling in EPCs. In vitro cell culture assays demonstrate that SDF-1 induces a positive feedback loop of its own receptor CXCR4 expression in EPCs through a non-canonical NF-κB signaling pathway. In addition, FGF23 could interfere with SDF-1-induced EPC senescence and ultimately conduce to improve AKI in SCID mouse model.

## Results

### FGF23 ameliorates kidney ischemia-reperfusion (IR) injury in mice

Following contralateral nephrectomy, unilateral ischemia and reperfusion (IR) in mice led to gradual increase of blood urea nitrogen (BUN) and serum creatinine (CRE) after reperfusion (Supplementary Fig. 1a, b). In addition, consistent with the FA-induced AKI mouse model^3^, intact FGF23 (iFGF23) levels began to rise significantly at 1 h after reperfusion (Supplementary Fig. 1c).

**Fig. 1:**
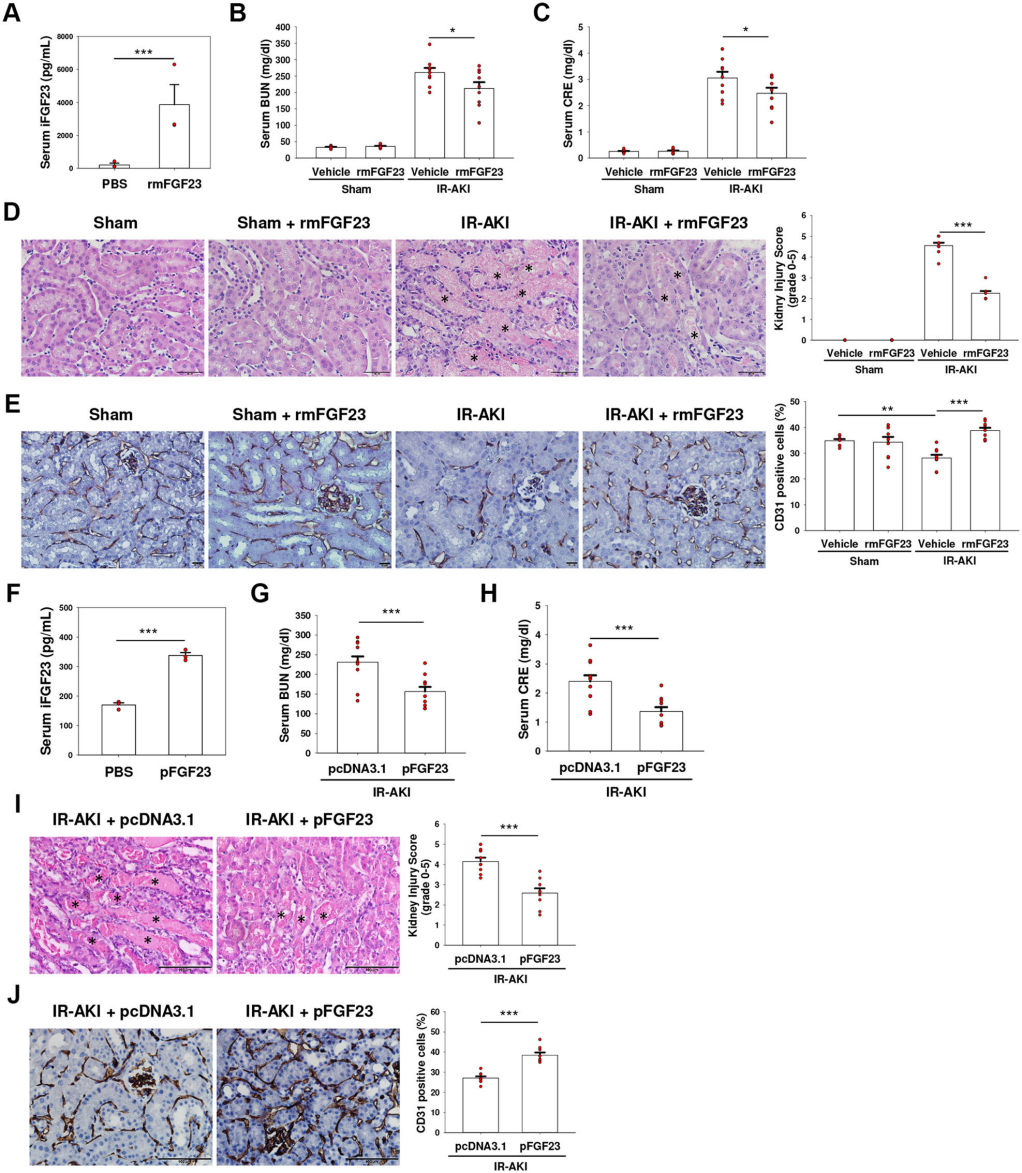
FGF23 ameliorates kidney ischemia-reperfusion (IR) injury in mice. **A**, **F** Plasma levels of iFGF23 were determined using ELISA kit after 30 min of FGF23 protein (**A**) or 24 h of pFGF23 plasmid (**F**) injection. Bars on graph are ±SEM (*n* = 3). ***p < 0.001 versus control mice by *t* test. **B**, **C** Recombinant mouse FGF23 (rmFGF23) were injected into C57BL/6 mice via tail-vein 30 min before IR injury. **G**, **H** FGF23 expression plasmid (pFGF23) was injected into C57BL/6 mice via tail-vein 24 h before IR injury. Serum blood urea nitrogen (BUN) (**B**, **G**) and creatinine (CRE) (**C**, **H**) were measured after 48 h of reperfusion. Bars on graphs are ± SEM. *p < 0.05 by *t* test, *n* = 9. **D**, **I** Kidneys were harvested after 48 h of reperfusion for H&E staining from FGF23 protein-injected (**D**) or pFGF23-injected mice (**I**). Necrotic tubules were marked with asterisks. Scale bars, 50 μm (**D**), 100 μm (**I**). Kidney injury score was graded using a semi-quantitative scale in ten random fields for each sample (*n* = 9). Bars on graph are ±SEM. ***p < 0.001 by *t* test. **E**, **J** Kidneys were harvested after 48 h of reperfusion immunohistochemistry with CD31 antibody from FGF23 protein-injected (**E**) or pFGF23-injected mice (**J**). Scale bars, 20 μm (**E**), 100 μm (**J**). The percentage of CD31 positive cells was counted in ten random fields for each sample (*n* = 9). Bars on graph are ±SEM. **p < 0.01, ***p < 0.001 by *t* test.

To generate a FGF23 gain-of-function mouse model, we directly injected the mutant cleavage-resistant form of recombinant mouse FGF23 into IR-AKI mice. After 30 min of injection, iFGF23 was increased in FGF23-injected mice compared with controls (Fig. 1A). Unexpectedly, serum levels of BUN and CRE in IR-AKI mice were ameliorated with FGF23 injection (Fig. 1B, C). Histology changes of AKI and AKI score were also attenuated (Fig. 1D).

Proliferation marker of PCNA and redifferentiation marker of E-cadherin were increased in FGF23-treated IR-AKI mice compared with IR-AKI mice (Supplementary Fig. 2a, c). TUNEL staining showed that cell death decreased with FGF23 injection (Supplementary Fig. 2d). Phosphorylation of Akt, cell survival signaling, was increased in FGF23-treated IR-AKI mouse compared with IR-AKI group (Supplementary Fig. 2a, b). Furthermore, CD31 immunostaining results showed that rarefaction of peritubular capillaries was noted in IR-AKI mouse kidneys. FGF23 injection into IR-AKI mice led to restored microvascular rarefaction (Fig. 1E).

In vivo FGF23-overexpressing IR-AKI model by plasmid delivery further confirmed these observations. After 24 h of plasmid injection, serum iFGF23 level was increased in pFGF23-injected mice compared with controls (Fig. 1F). Similar results were obtained (Fig. 1G–J), indicating that FGF23 may play a pivotal protective role in renal microvascular damage and acute kidney IR injury.

### FGF23 inhibits SDF-1-induced CXCR4 expression in EPCs

**Fig. 2:**
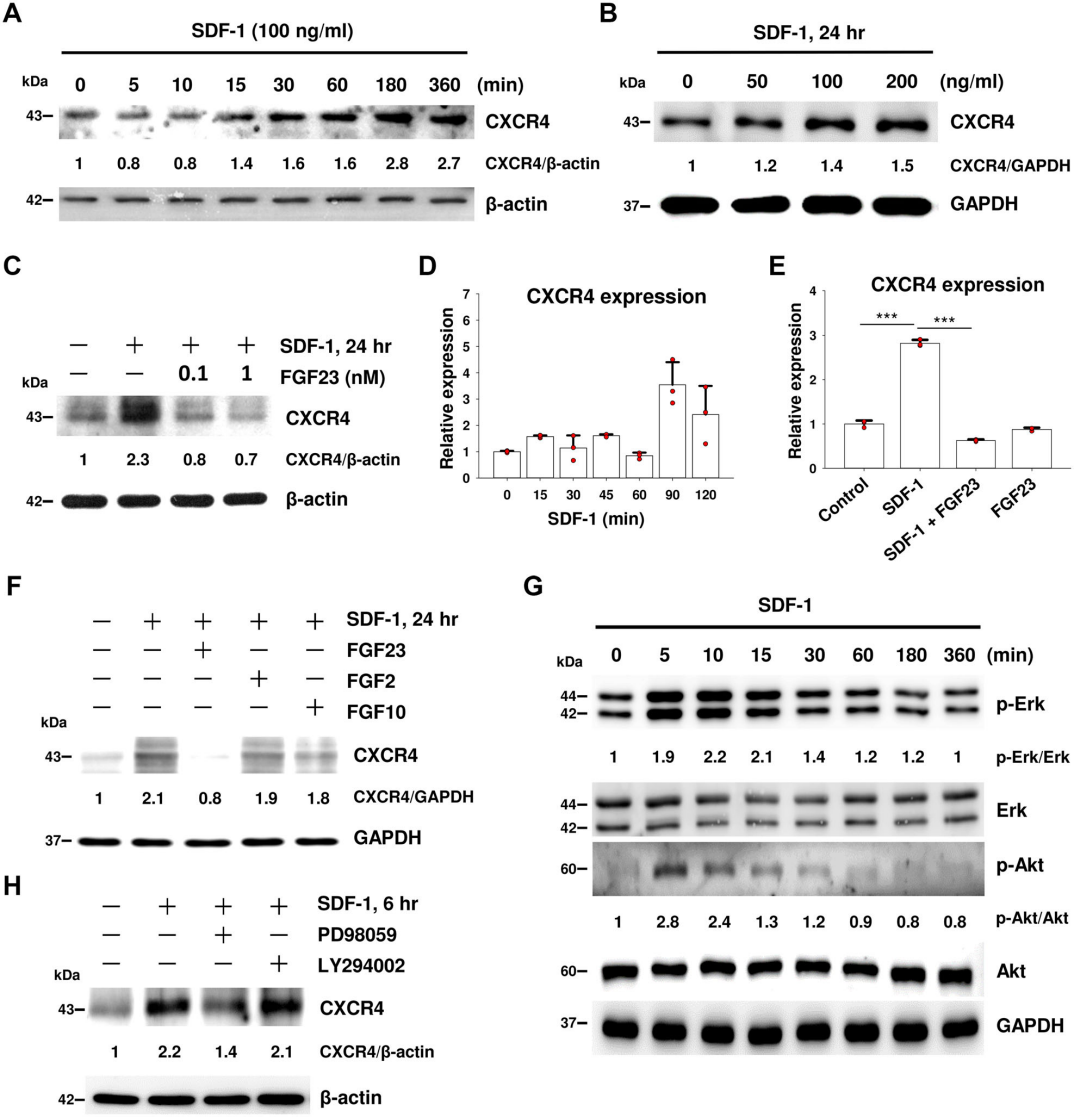
FGF23 inhibits SDF-1-induced CXCR4 expression in EPCs. **A** EPCs were collected at the indicated time points after SDF-1 treatment and immunoblotted with CXCR4 antibody. **B** EPCs were treated with different doses of SDF-1 and immunoblotted with CXCR4 antibody. **C** EPCs were pre-treated with different doses of FGF23 for 30 min before SDF-1 stimulation. CXCR4 expression was detected by western blot. **D**, **E** EPCs were treated with SDF-1 at the indicated time points (**D**). EPCs were pre-treated with FGF23 for 30 min followed by treatment of SDF-1 for 90 min (**E**). CXCR4 mRNA expression level was shown by q-PCR, and normalized to actin. Bars on graphs are ±SD. ***p < 0.001 by *t* test, *n* = 3. **F** EPCs were pre-treated with FGF23, FGF2, or FGF10 for 30 min followed by SDF-1 stimulation for 24 h. CXCR4 expression was detected by western blot. **G** EPCs were collected at the indicated time points after SDF-1 treatment and immunoblotted with p-Erk, Erk, p-Akt, and Akt antibodies. **H** EPCs were pre-treated with Erk inhibitor (PD98059) or Akt inhibitor (LY294002) for 30 min before SDF-1 stimulation. CXCR4 expression was detected by western blot. GAPDH or β-actin were used as protein loading controls. The numbers under the gel lanes represent the relative protein level.

As Injecting FGF23 mitigated microvascular rarefaction in AKI mice, we explored the role of FGF23 in human umbilical cord blood-derived late EPCs (Supplementary Fig. 3). We showed that SDF-1 induced time- and dose-dependent expression of CXCR4 protein (Fig. 2A, B). SDF-1-induced CXCR4 protein expression was inhibited by FGF23 (Fig. 2C). SDF-1-induced CXCR4 mRNA transcript was augmented time-dependently and suppressed by FGF23 (Fig. 2D, E). We further tested whether SDF-1-mediated CXCR4 expression is also inhibited by these FGF ligands. Our results showed that the response is specific for FGF23 (Fig. 2F).

Phosphorylation of Akt and Erk by SDF-1 were significantly increased at 5–15 min (Fig. 2G). Pretreatment with the Erk inhibitor (PD98059), but not the Akt inhibitor (LY29402), inhibited SDF-1-induced CXCR4 expression (Fig. 2H). These data indicate that phosphorylation and activation of Erk regulates SDF-1-mediated CXCR4 expression.

### SDF-1 induces CXCR4 expression via the non-canonical NF-κB signaling pathway in EPCs

Western blot showed that a time-dependent phosphorylation of RelA was induced by SDF-1. However, we could not observe any activation of canonical NF-κB signaling, including IKKα, IKKβ, and IκB proteins after SDF-1 stimulation (Supplementary Fig. 4a, b). Ribosomal S6 kinases (RSK) phosphorylation, a regulator of the non-canonical NF-κB signaling, was increased in a time-dependent manner at residue Ser380, but not at Thr359 or Thr573 (Supplementary Fig. 4c). Furthermore, FGF23 attenuated SDF-1-induced Erk/RSK/RelA phosphorylation and CXCR4 expression (Supplementary Fig. 4d).

### RelA acts as a transcriptional repressor of CXCR4 gene in EPCs

**Fig. 3:**
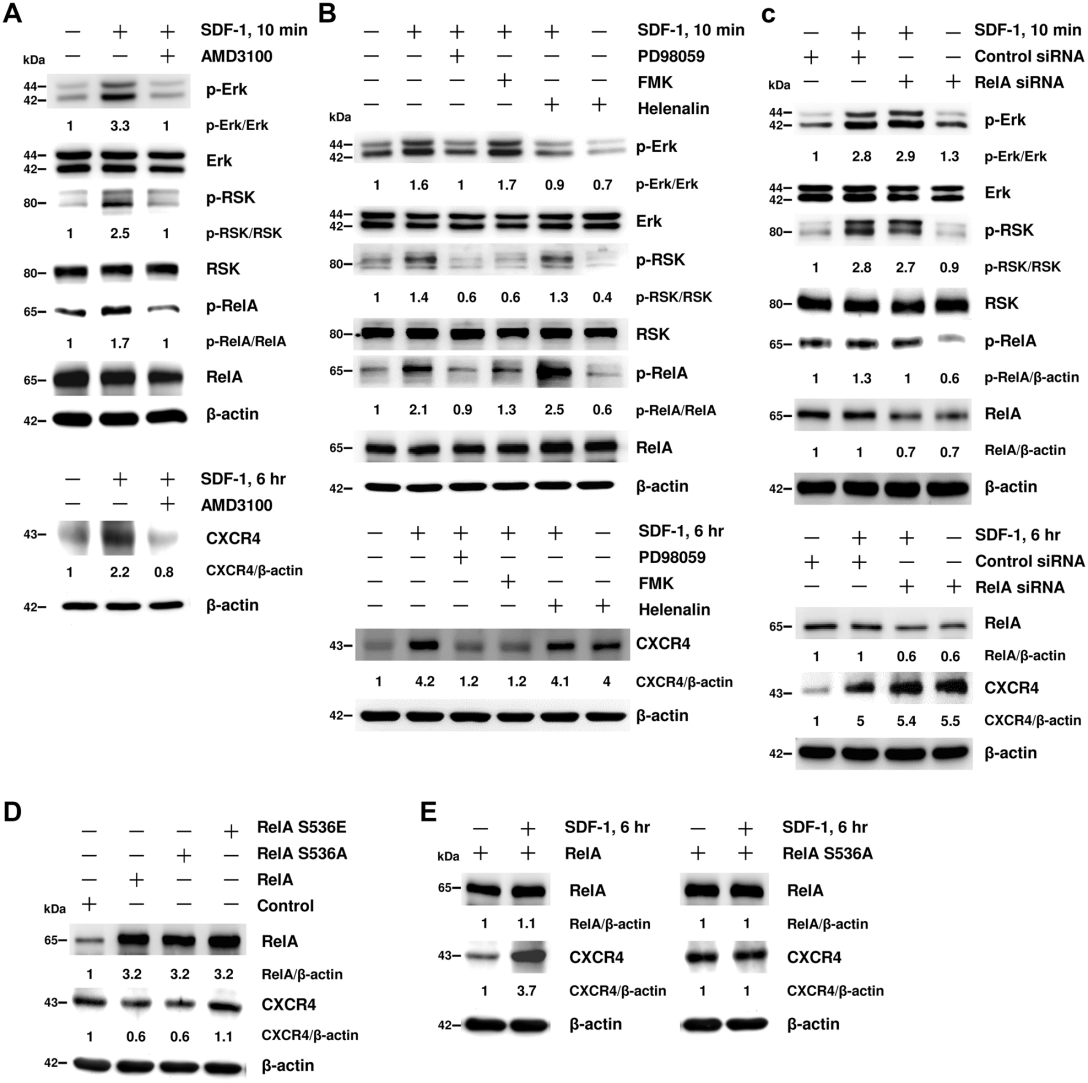
RelA conducts as a transcriptional repressor of CXCR4 gene in EPCs. **A** EPCs were pre-treated with CXCR4 inhibitor (AMD3100) for 30 min followed by SDF-1 stimulation for 10 min or 6 h. **B** EPCs were pre-treated with Erk inhibitor (PD98059), RSK inhibitor (FMK), or NF-κB inhibitor (Helenalin) for 30 min followed by SDF-1 stimulation for 10 min or 6 h. **C** EPCs harboring RelA or control siRNA were treated with SDF-1 for 10 min or 6 h. **D** WT, non-phosphorylatable S536A, or phospho-mimicking S536E mutant RelA were overexpressed in EPCs. **E** WT or non-phosphorylatable S536A RelA were overexpressed in EPCs followed by SDF-1 stimulation for 6 h. Protein lysates were analyzed by immunoblot using indicated antibodies. β-actin was used as a protein loading control. The numbers under the gel lanes represent the relative protein level.

Pretreatment of CXCR4 inhibitor (AMD3100) (Fig. 3A) and Erk inhibitor (PD98059) suppressed Erk/RSK/RelA phosphorylation as well as CXCR4 expression (Fig. 3B). Pretreatment of RSK inhibitor (FMK) reduced RelA phosphorylation and CXCR4 expression, but not Erk phosphorylation.

However, an unexpected finding was that CXCR4 expression was augmented when EPCs were pre-treated with RelA inhibitor (Helenalin) (Fig. 3B), which prevent RelA from binding to DNA^22^. By inference, this data implies that RelA might act as a transcriptional repressor for CXCR4 gene. As expected, knockdown of RelA by siRNA augmented CXCR4 expression without changes in Erk and RSK phosphorylation (Fig. 3C).

To confirm whether phosphorylation at serine residue S536 is critical for RelA to regulate CXCR4 expression, we overexpressed WT, S536A, or S536E RelA in EPCs. Overexpression of WT RelA or non-phosphorylatable S536A mutant decreased CXCR4 expression, whereas phosphomimetic S536E mutant did not (Fig. 3D). Exposure of EPCs harboring WT RelA to SDF-1 resulted in an augmented CXCR4 expression, but EPCs expressing non-phosphorylatable S536A mutant did not (Fig. 3E). Our results delineated that RelA played a negative regulatory role in CXCR4 expression, and the phosphorylation at RelA Ser536 played an important part in CXCR4 transcription.

### SDF-1 induces the export of basal nuclear NF-κB (RelA) from nucleus

In normal EPCs, scattered signals of the Erk were detected throughout the cell, whereas RSK and RelA were sequestered in the nucleus. After SDF-1 stimulation, dense signals of Erk, p-Erk, RSK, and p-RSK were detected mainly in the nucleus. Moreover, RelA showed a diffuse cytosolic distribution rather than nuclear accumulation upon treatment with SDF-1 and adding FGF23 led to increased RelA transition from cytosolic distribution to nuclear accumulation. Notably, p-RelA was cytosolic and accumulated around the nucleus; however, p-RelA did not redistrubute after SDF-1 and/or FGF23 treatment (Supplementary Fig. 5a). Western blot analysis showed that RelA and p-RelA decreased in the nuclear fraction after SDF-1 treatment, while FGF23 could counteract the SDF-1 effect (Supplementary Fig. 5b). Incubation of SDF-1-treated EPCs with leptomycin B (LMB), a specific inhibitor of nuclear export, could enhance nuclear accumulation of RelA (Supplementary Fig. 5c). These results indicated that cytosolic accumulation of RelA in SDF-1-treated EPCs was due to translocating RelA from the nucleus to the cytoplasm rather than preventing import of RelA into nucleus. Taken together, our results revealed that SDF-1 induced Erk phosphorylation and translocation of p-Erk from the cytoplasm into the nucleus, leading to nuclear RSK phosphorylation. Furthermore, basal nuclear RelA was phosphorylated by p-RSK and exported from the nucleus to the cytoplasm. FGF23 could abrogate SDF-1-induced Erk/RSK/RelA signaling pathway via preventing Erk phosphorylation and translocation.

### SDF-1 prevents RelA binding to CXCR4 promoter in vitro and in vivo

**Fig. 4:**
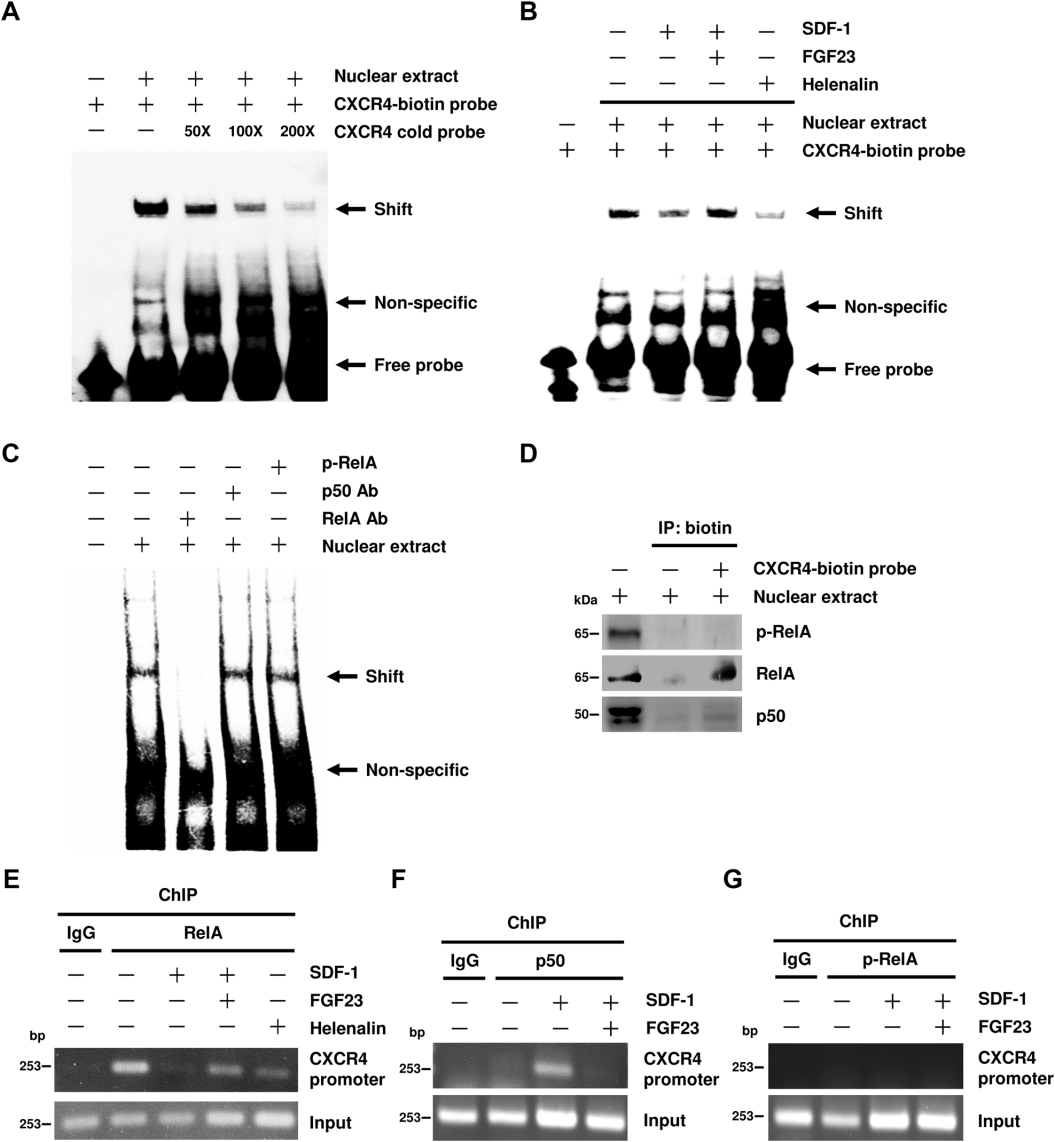
SDF-1 inhibits RelA binding to CXCR4 promoter in vitro and in vivo. **A** Nuclear protein extracts were prepared from untreated EPCs and analyzed by EMSA with the CXCR4-biotin probe. Specific competition was performed with the 50-, 100- or 200-fold molar excess of unlabeled CXCR4 probe. **B** EPCs were pre-treated with FGF23 for 30 min followed by SDF-1 stimulation for 10 min. Treatment of NF-κB inhibitor (Helenalin) for 40 min was used as a positive control. Nuclear protein extracts were prepared and analyzed by EMSA with the CXCR4-biotin probe. **C** For supershift assay, RelA, p-RelA, or p50 Antibodies were incubated with untreated EPC nuclear extracts prior to the addition of the CXCR4-biotin probe. **D** For DAPA assay, untreated EPC nuclear extracts were incubated with the CXCR4-biotin probe and streptavidin beads. The beads were further analyzed by western blotting with RelA, p-RelA, or p50 Antibodies. **E**–**G** EPCs were pre-treated with FGF23 for 30 min followed by SDF-1 stimulation for 15 min. Incubation of NF-κB inhibitor (Helenalin) for 40 min was used as a positive control. For ChIP assays, cross-linked DNA were immunoprecipitated with RelA (**E**), p50 (**F**), or p-RelA (**G**) antibodies and subjected to PCR with CXCR4 specific primers. Treatment with IgG antibody was used as a negative control. PCR product of unprecipitated DNA was used as an input control.

Our result revealed that RelA binds to CXCR4 promoter in control EPC nuclear extracts using electrophoretic mobility shift assay (EMSA). The competing cold probe could eliminate the shifted band (Fig. 4A). The shifted band was also abolished after EPCs were treated with SDF-1 or Helenalin. FGF23 counteracted this SDF-1-mediated effect. (Fig. 4B). Incubation with anti-RelA, but not with anti-p50 or anti-p-RelA antibodies, dissipated the specific shift band, indicating that only RelA is involved in this DNA–protein complex (Fig. 4C). DNA affinity precipitation assay (DAPA) also showed that CXCR4-biotin probe only pulled down RelA protein, but not p50 or p-RelA (Fig. 4D). In vivo chromatin immunoprecipitation (ChIP) assay showed that RelA significantly bound to the CXCR4 promoter in EPCs, however, p50 did not. Helenalin diminished the recruitment of RelA to the CXCR4 promoter. Once EPCs were exposed to SDF-1, RelA was phosphorylated and then released from the CXCR4 promoter; however, p50 could be recruited to the CXCR4 promoter. Furthermore, FGF23 counteracted the effect of SDF-1 treatment (Fig. 4E, F). In contrast, we did not find p-RelA bound to the CXCR4 promoter in control, SDF-1 and/or FGF23-treated EPCs (Fig. 4G).

### SDF-1 induces CXCR4 expression through inhibition of GLP-mediated epigenetic gene repression

RelA has been shown to cause DNA methylation-based epigenetic repression of downstream gene expression via interaction with euchromatic histone-lysine N-methyltransferase 1 (EHMT1/GLP)^23^. Western blot results showed that CXCR4 expression was elevated in EPCs after GLP inhibitor (UNC0224) treatment (Supplementary Fig. 6a). The co-immunoprecipitation experiment showed that RelA interacted with GLP in EPCs (Supplementary Fig. 6b). Importantly, the ChIP data showed that GLP was recruited to the CXCR4 promoter in control EPCs (Supplementary Fig. 6c). A previous study has shown that the dimethylated state of histone H3 at lysine residue 9 (H3K9me2) by G9a/GLP is associated with transcriptional repression of genes^24^. As anticipated, we found that H3K9me2 was highly enriched in control EPCs (Supplementary Fig. 6d). GLP recruitment was compromised with consequent decrease in H3K9me2 levels after SDF-1 treatment (Supplementary Fig. 6c, d). In contrast, the interaction of the RelA with the general co-activator protein p300 did not relate to CXCR4 promotor (Supplementary Fig. 6e). SDF-1-mediated effects were abolished by FGF23 pretreatment (Supplementary Fig. 6c–e). These data suggested that GLP was recruited to the CXCR4 promoter to interact with RelA, and the GLP-RelA complex further catalyzed H3K9 dimethylation to suppress CXCR4 expression. SDF-1 induced RelA phosphorylation and prevented the recruitment of GLP, dimethylation of histone H3K9, and thus induced CXCR4 expression (Supplementary Fig. 8).

### RelA acts as a transcriptional activator of angiogenic cytokines in EPCs

Several pro-angiogenic cytokines such as IL-6, IL-8, and VEGF-A are targets of NF-κB and released by EPCs^25–27^. We evaluated whether SDF-1-mediated RelA signaling also regulates these cytokines in EPCs. VEGF-A, IL-6, and IL-8 promoters were bound by RelA in untreated EPCs using ChIP assay (Supplementary Fig. 7a). Additionally, p50 was recruited to IL-8 promoter (Supplementary Fig. 7b), but not to VEGF-A, IL-6 or CXCR4 promoter. Treatment of SDF-1 released RelA–RelA and RelA–p50 complexes from the promoter regions of these cytokines, while consequent pretreatment with FGF23 counteracted the effects of SDF-1 (Supplementary Fig. 7a, b).

We next evaluated the effects of SDF-1 and/or FGF23 on EPC production of these cytokines. VEGF-A, IL-6, and IL-8 mRNA transcripts, and protein levels in culture supernatants were suppressed after SDF-1 treatment. SDF-1-inhibited cytokine production were counteracted by FGF23 (Supplementary Fig. 7c, d). Results of ChIP analysis showed that p300 bound to the promoter of the VEGF-A and IL-6 genes. After SDF-1 treatment, binding of p300 to these promoters were inhibited. The presence of FGF23 blocked the ability of SDF-1 to induce p300 dissociation (Supplementary Fig. 7g). GLP binding and H3K9 dimethylation did not change at the VEGF-A and IL-6 promoters following SDF-1 and/or FGF23 treatment (Supplementary Fig. 7e, f). In contrast, the recruitment of p300 to the IL-8 promoter was not apparent after treatments (Supplementary Fig. 7g). Rather, GLP binding and H3K9 dimethylation were increased at the IL-8 promoter after SDF-1 treatment. FGF23 abolished this SDF-1-induced GLP binding and H3K9 dimethylation at the IL-8 promoter (Supplementary Fig. 7e, f). Taken together, SDF-1 downregulated VEGF-A and IL-6 expression through inhibition of co-activator protein p300 recruitment, whereas it downregulated IL-8 expression through enhancement of GLP binding and H3K9 dimethylation (Supplementary Fig. 8).

### FGF23 attenuates SDF-1-induced CXCR4 expression via interaction of FGF receptor-1 (FGFR1), but not Klotho

**Fig. 5:**
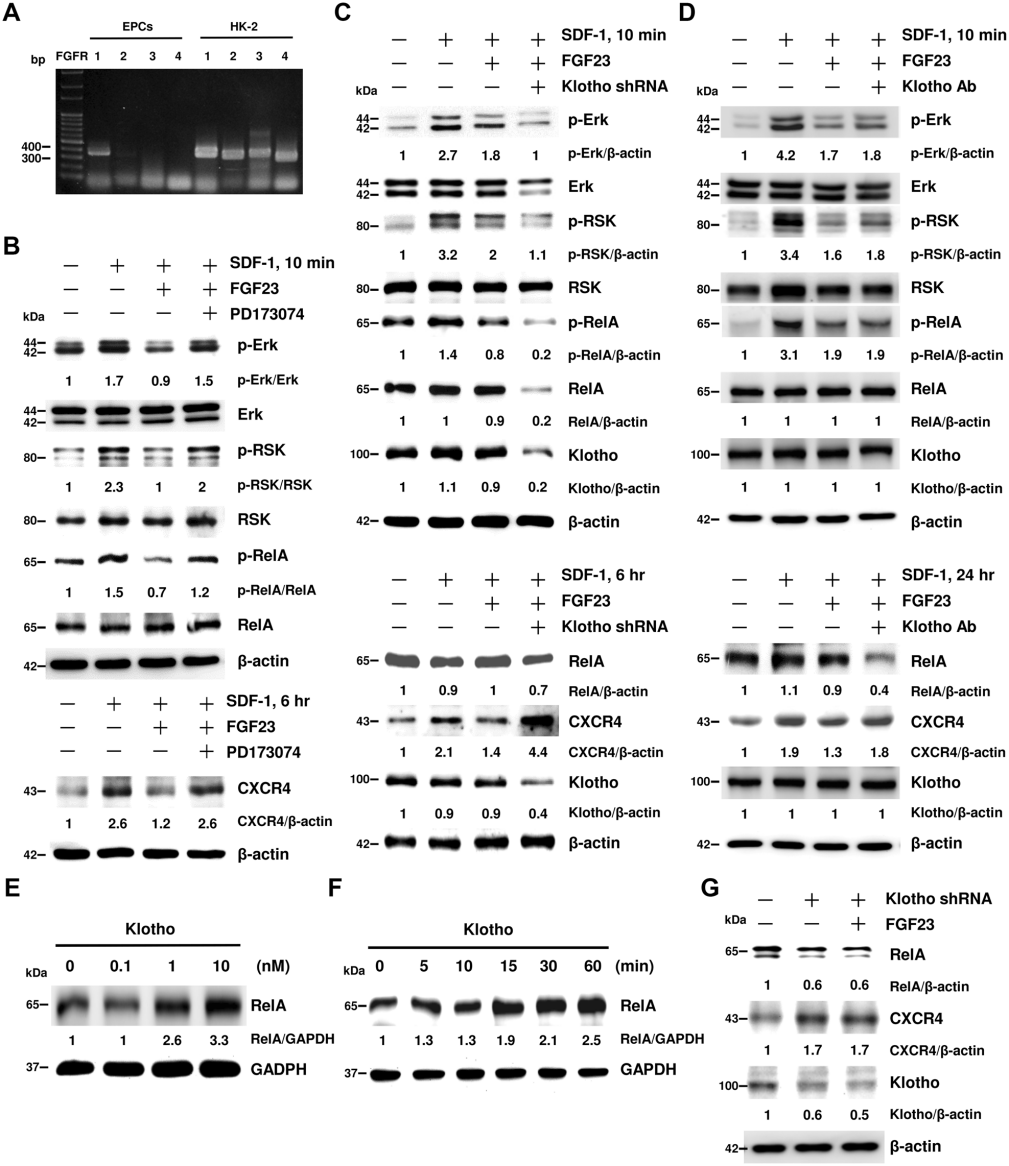
FGF23 attenuates SDF-1-induced CXCR4 expression via interaction of FGF receptor-1 (FGFR1), but not Klotho. **A** FGFR-1, -2, -3, or -4 mRNA expression levels in EPCs were detected by RT-PCR. HK-2 lysates were used as positive controls. **B** EPCs were pre-treated with FGF23 and/or FGFR inhibitor (PD173074) for 30 min followed by SDF-1 stimulation for 15 min or 6 h. **C** EPCs harboring control or Klotho shRNA were pre-treated with FGF23 for 30 min followed by SDF-1 stimulation for 10 min or 6 h. **D** EPCs were pre-treated with Klotho neutralizing antibody for 60 min and FGF23 for 30 min followed by SDF-1 stimulation for 10 min or 24 h. **E** EPCs were treated with different doses of Klotho. **F** EPCs were collected at the indicated time points after Klotho treatment. **G** EPCs harboring control or Klotho shRNA were pre-treated with FGF23 for 24 h. Protein lysates were analyzed by immunoblot using indicated antibodies. β-actin or GAPDH were used as protein loading controls. The numbers under the gel lanes represent the relative protein level.

The mechanism by which FGF23 suppresses SDF-1-induced CXCR4 expression has not been elucidated. In EPCs, only FGF receptor-1 (FGFR1) transcript was expressed, but not FGFR2–4 (Fig. 5A). FGF23-inhibited Erk/RSK/RelA phosphorylation and CXCR4 expression were completely reversed by pan-FGFR inhibitor (PD173074) (Fig. 5B). To further verify whether FGF23 co-receptor Klotho also participates in the regulation of FGF23 signaling, we used short hairpin (sh)RNA to knockdown Klotho. Interestingly, FGF23-inhibited CXCR4 expression was reversed by knockdown of Klotho; however, FGF23-inhibited Erk/RSK/RelA phosphorylation were not (Fig. 5C). Another noteworthy finding is that knockdown of Klotho resulted in downregulation of RelA protein, implying that the dramatic increase CXCR4 expression in Klotho knockdown EPCs was caused by loss of RelA protein, rather than modulation of Erk/RSK/RelA phosphorylation (Fig. 5C). Because long-term knockdown of Klotho by shRNA resulted in the unexpected loss of RelA protein, we next used a neutralizing antibody to block Klotho activity. Our results showed that neither the amount of RelA protein nor phosphorylation of Erk/RSK/RelA was changed by short-term neutralizing of Klotho activity (Fig. 5D, upper panel), whereas, after 24 h of Klotho neutralizing antibody treatment, RelA protein was downregulated, which further resulted in CXCR4 overexpression (Fig. 5D, lower panel). To further confirm whether Klotho indeed regulates RelA protein expression, EPCs were treated with recombinant soluble Klotho (sKlotho). RelA protein levels were upregulated by sKlotho in dose- and time-dependent manners (Fig. 5E, F). Moreover, upregulation of CXCR4 by Klotho knockdown could not be suppressed by FGF23 treatment (Fig. 5G). Our findings indicated that FGFR1, but not Klotho, was indispensable for activation of FGF23 signaling in EPCs. Blocking of Klotho did not interfere with the signaling transduction of FGF23-FGFR1 complexes; however, RelA protein, which is a downstream component of FGF23 signaling in EPCs, was regulated by Klotho.

### SDF-1-mediated EPC migration and senescence, but not angiogenesis, are suppressed by FGF23

**Fig. 6:**
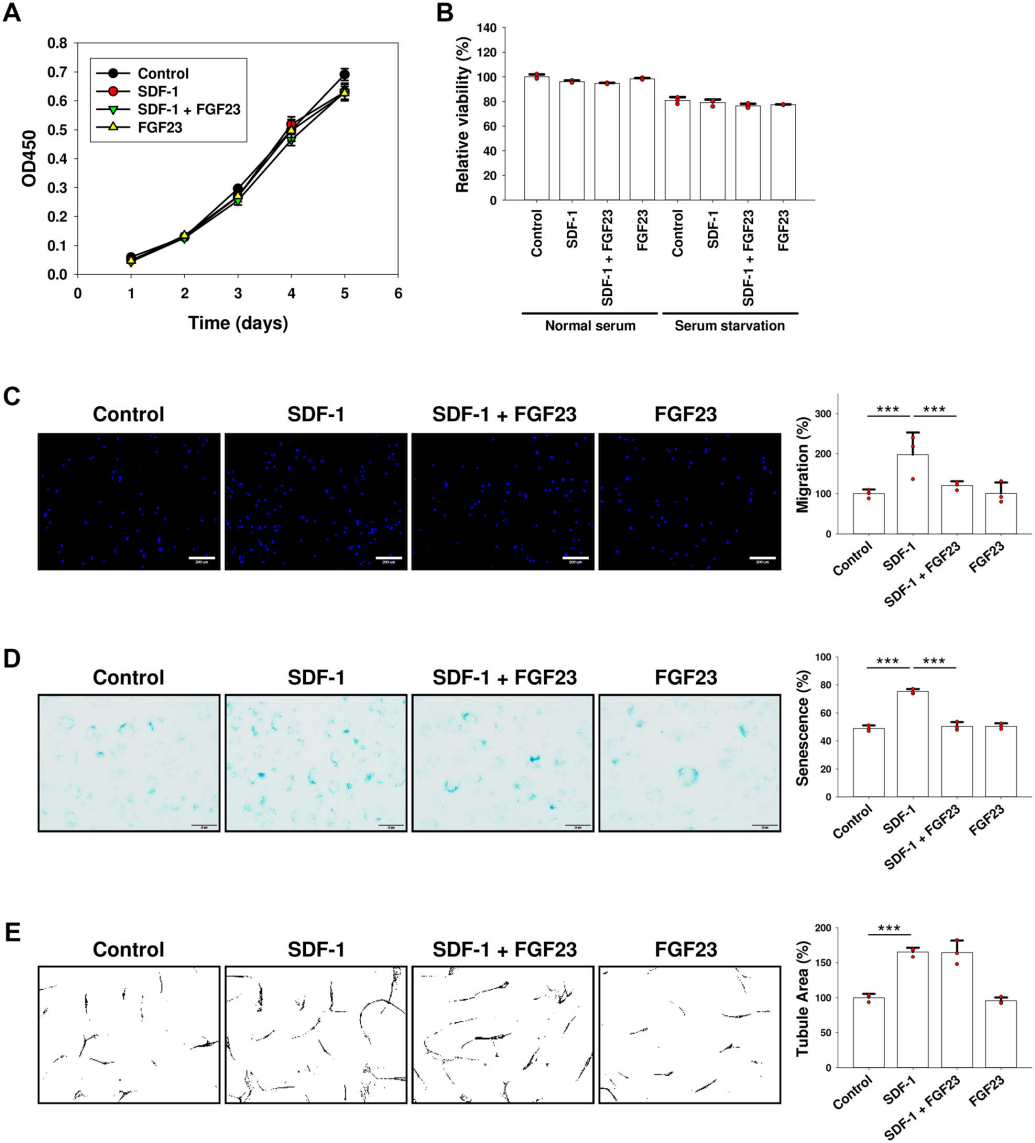
SDF-1-mediated EPC migration and senescence are suppressed by FGF23, but not angiogenesis. **A** EPCs were treated with FGF23 and/or SDF-1 for 5 days. Proliferation was evaluated by CCK-8 assay. Bars on graphs are ±SD, *n* = 3. **B** Serum-starved and control EPCs were treated with FGF23 and/or SDF-1 for 2 days. Cell viability was measured by CCK-8 assay. Bars on graphs are ±SD, *n* = 3. **C** EPC migration was detected by a modified Boyden chamber assay. EPCs were treated with SDF-1 and/or FGF23 for 24 h, and reseeded in the upper compartment. And then SDF-1 was placed in the lower compartment to attract EPC migration for 3 h. Migrated EPCs were stained with Hoechst 33342 and counted in six random fields for each sample (*n* = 3). Scale bars, 200 μm. Bars on graphs are ±SD. ***p < 0.001 by *t* test. **D** EPCs were treated with SDF-1 and/or FGF23 for 5 days, and stained with β-gal. The percentage of senescent cells was counted in ten random fields for each sample (*n* = 3). Scale bars, 50 μm. Bars on graphs are ±SD. ***p < 0.001 by *t* test. **E** EPCs were cultured on MRC-5 feeder layers, and treated with SDF-1 and/or FGF23 for 6 days. The endothelial cells were verified by IHC using CD31 antibody. The resulting images were then converted to binary images. The percentage of capillary morphogenesis was counted in ten random fields for each sample (*n* = 3; HPF, 100×). Bars on graphs are ±SD. ***p < 0.001 by *t* test.

We further assessed whether in vitro cellular functions of EPCs were altered by SDF-1 and/or FGF23 treatment. We observed no difference in cell proliferation and death between the experimental and control cells (Fig. 6A, B). SDF-1 induced EPC migration and senescence, and FGF23 attenuated those effects (Fig. 6C, D). CD31 immunostaining results showed that SDF-1 enhanced EPC angiogenesis in vitro. Notably, FGF23 played no role in EPC angiogenesis (Fig. 6E). Taken together, our data showed that migration, senescence, and angiogenesis of EPCs were enhanced by SDF-1 in vitro; however, FGF23 blocked SDF-1-induced migration and senescence, but not angiogenesis.

### FGF23 reverses SDF-1-induced impaired therapeutic potential of EPCs in IR-AKI SCID mice

**Fig. 7:**
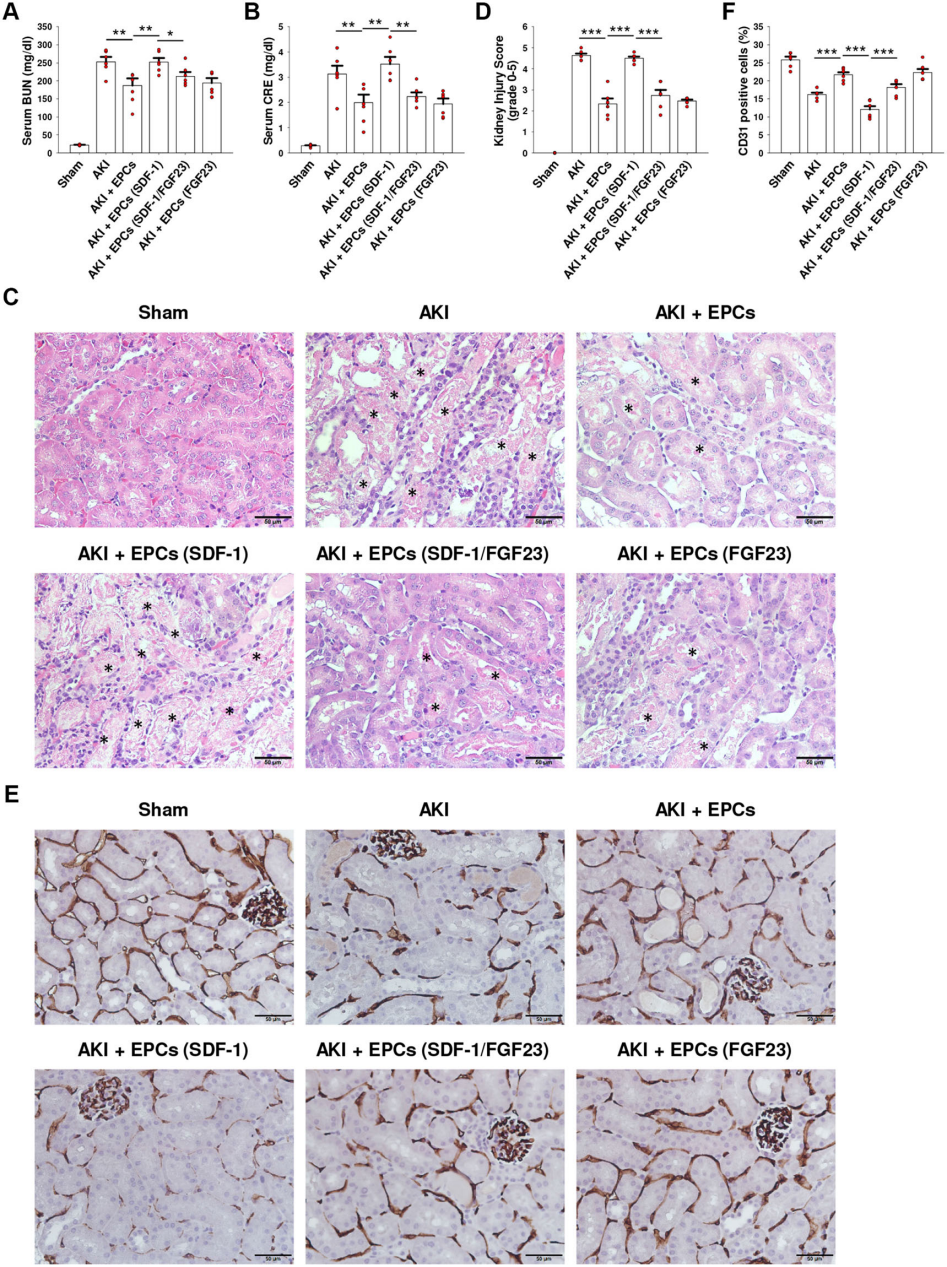
FGF23 reverses SDF-1-induced impaired therapeutic potential of EPCs in severe combined immunodeficiency (SCID) mice after ischemia-reperfusion (IR) injury. **A**, **B** EPCs were pre-treated with FGF23 and/or SDF-1 ex vivo for 24 h and injected into SCID mice via tail-vein 30 min before IR injury. Serum blood urea nitrogen (BUN) (**A**) and creatinine (CRE) (**B**) were measured after 48 h of reperfusion. Bars on graphs are ±SEM. *p < 0.05, **p < 0.01 by *t* test, *n* = 6. **C**, **D** Kidneys were harvested after 48 h of reperfusion for H&E staining (**C**). Necrotic tubules were marked with asterisks. Scale bars, 50 μm. Kidney injury score was graded using a semi-quantitative scale in ten random fields for each sample (*n* = 6) (**D**). Bars on graph are ±SEM. ***p < 0.001 by *t* test. **E**, **F** Kidneys were harvested after 48 h of reperfusion and subjected to IHC with CD31 antibody (**E**). Scale bars, 50 μm. The percentage of CD31 positive cells was counted in ten random fields for each sample (*n* = 6) (**F**). Bars on graph are ±SEM. ***p < 0.001 by *t* test.

To validate whether FGF23 and SDF-1 could affect the therapeutic ability of EPCs in vivo, control, SDF-1-treated, FGF23-treated, and FGF23 plus SDF-1 co-incubated EPCs were injected into the tail vein of the IR-AKI SCID mice. At 48 h after ischemia-reperfusion, serum BUN and CRE levels were increased in IR-AKI SCID mice. Intravenous injection of untreated EPCs after ischemia-reperfusion reduced BUN and CRE levels. The therapeutic ability of EPCs was attenuated by ex vivo pretreatment of EPCs with SDF-1. Interestingly, the attenuated therapeutic ability of SDF-1-treated-EPCs was restored by ex vivo co-incubation with FGF23 (Fig. 7A, B). Severe acute tubular necrosis, luminal casts, and microvascular rarefaction occurred in AKI and AKI + SDF-1-treated-EPCs mice. Injured renal tubules and capillaries were restored by injection of EPCs, SDF-1/FGF23-co-treated-EPCs, and FGF23-treated-EPCs (Fig. 7C–F).

## Discussion

Our data showed that exogenous FGF23 administration results in increased tubular cell regeneration, reduced cell death and microvascular rarefaction in the AKI kidneys. Treatment with EPCs attenuates the kidney injury of AKI SCID mice; ex vivo pretreatment of EPCs with SDF-1 abrogates the therapeutic effect of EPCs that were injected into AKI SCID mice, but it was rescued by the presence of ex vivo FGF23. We dissected the cellular and molecular mechanisms underlying the therapeutic effect of FGF23, and demonstrated a klotho-independent, causal role for FGF23 in the suppression of SDF-1/CXCR4 via inhibition of a non-canonical Erk/RSK/NF-κB phosphorylation, and suggested that FGF23 contributes to the decrease of SDF-1-induced senescence of EPCs as a contributor for renoprotection during AKI (Supplementary Fig. 9). Our findings provide the experimental evidence to verify the pathophysiological role of FGF23 in AKI, and may give us more insight for developing effective approaches to rescue AKI.

Accumulating evidence has shown that high circulating levels of FGF23 is associated with adverse outcomes in AKI. Thus, elevated FGF23 level traditionally seemed not only a biomarker but also a nephrotoxic culprit for pessimistic development of AKI. However, the prior study showed that the poor CKD outcomes, increased serum level of BUN and mortality^28^ after depletion of FGF23 with FGF23 antibody stimulated rethinking the pathophysiological role of FGF23^29^. With a mouse unilateral ureteral obstruction (UUO) model, injection of FGF23 does not exacerbate renal failure^30^. These data seem to infer that FGF23 is not as detrimental as previously anticipated. Collectively, it still remains a matter of debate because of no experimental evidence had ever demonstrated the real pathophysiological role of FGF23 in AKI until now. Here, our results showed that FGF23 plays an unexpected protective role in a murine model of IR-AKI, and diminished therapeutic ability of SDF-1-treated EPCs is counteracted by FGF23.

Our data showed that exogenous FGF23 administration results in increased proximal tubular cell regeneration and reduced cell death in the outer stripe of AKI kidneys, and eventually ameliorates AKI. Of note, while our evidence indicated that FGF23 ameliorates AKI via modulation of EPCs, the other group has reported that FGF23-Klotho signaling pathway could enhance proliferation and inhibit vitamin D-induced apoptosis in renal proximal tubular epithelial cells (PTECs)^31^ and FGF23 might exert its beneficial effects directly on renal tubular cells. However, our in vitro investigation on whether FGF23, in combination with or without Klotho protein, exerts its beneficial effects directly on renal tubular cells did not show any significant positive findings (data not shown). Therefore, whether FGF23 also has direct protective effects on proximal tubular epithelial cells need further study.

Inflammation is now believed to play a major role in the pathophysiology of AKI, leading to the extension phases of injury. Prevention of renal neutrophil recruitment during AKI has been shown to result in protection of renal function^32–35^. Recently, an elegant study has demonstrated that FGF23 could impair neutrophil recruitment during chronic renal disease (CKD) through counteraction of integrin activation on neutrophils^36^. However, it is still unknown whether FGF23 could attenuate neutrophil recruitment during AKI, and whether this effect could contribute to ameliorating AKI.

Most importantly, although we could not entirely exclude the possibility that FGF23 has direct effects on renal tubular cells and/or neutrophils to ameliorate AKI at this moment, at least our data provide convincing evidence that EPCs play a critical role in the response to FGF23 treatment in in vivo and vitro models.

We demonstrated that SDF-1 prompts EPC migration, senescence, and differentiation in vitro, whereas FGF23 attenuates SDF-1-induced migration and senescence, but not angiogenesis. Traditionally, the CXCR4 protein levels and in vitro migration ability of EPCs were used to represent the therapeutic capacity of EPCs in vivo^37,38^. Intriguingly, although SDF-1-inducd EPCs express much more CXCR4 protein and show much higher migration activity than untreated controls in vitro, our results revealed that the in vivo therapeutic capacity of EPCs is eliminated by SDF-1, but restored by the concomitant addition of FGF23. One possibility for the lack of a much stronger correlation between migration ability and therapeutic capacity could be due to the senescence of SDF-1-induced EPCs. Apart from this, another possibility is that the migration ability of EPCs is not a key factor in the regeneration process during AKI. An elegant study recently demonstrated that recruitment of EPCs from an extrarenal EPC niche to the kidney during AKI is dispensable for kidney regeneration. It has never been shown that extrarenal EPCs actually integrate into the renal microvasculature and differentiate into mature endothelial cells to facilitate repair^39^. Recent evidence also indicates that the number of incorporated EPCs into ischemic tissue is quite low, thus the paracrine hypothesis of EPC in rescuing AKI has become more plausible^25,27^. Our data also showed that even though injection of human EPCs ameliorated the IR-AKI of SCID mice, no obvious numbers of human EPCs were detected and observed in the kidney of those SCID mice after EPC injection using human-specific antibody (data not shown). A recent study by Burger et al. has demonstrated therapeutic results which coincided with our observation^40^. They also used the same cell type as our human umbilical cord-derived late EPCs. They found that most of the EPCs are immediately detected in the lungs, but rare in the kidneys after injection. After 24 h, EPCs are barely detectable in any tissues. However, injection of microvesicles derived from EPC medium is sufficient to exert its protective effects in IR-AKI mice via paracrine effect. Given all these observations, we therefore emphasize again that we should be skeptical about using CXCR4 protein levels and in vitro migration ability to represent the therapeutic potential of EPCs in the future.

Under pathological conditions, SDF-1 induces EPC migration to the injury site and differentiation into mature endothelial cells^41^. A previous study has shown that SDF-1 analogue could prevent TNF-α-induced EPC senescence in vitro^42^. On the contrary, our data show that SDF-1 treatment alone promotes EPC senescence in vitro. One possibility is that our in vitro cell culture condition is likely to be similar to the physiological situation without any injury stimuli. Under physiological conditions, CXCR4-expressing EPCs are tethered predominantly in a specific niche in the bone marrow, where they interact with SDF-1-expressing stromal cells^43^. As mentioned above, SDF-1/CXCR4 signaling also induces EPC differentiation into mature endothelial cells, which loss of self-renewal capacity and those differentiated EPCs are more prone to senescence. Regardless, the overarching question remains: how could EPCs maintain their stem cell properties without undergoing senescence in SDF-1-enriched bone marrow niche? Data presented herein clearly show that FGF23 prevents SDF-1-induced senescence in vitro and restores insufficient therapeutic ability of SDF-1-treated-EPCs in vivo. Therefore, our data appear to imply that FGF23 might play a critical role in the maintenance of critical EPC properties in physiological condition.

NF-kB transcription factors act as a central mediator in inflammation, immunity, infections, proliferation, differentiation, and apoptosis^44–50^. The traditional ‘canonical pathway’^51^ shows the majority of NF-κB complexes are sequestered in the cytoplasm as an inactive form by interaction with its inhibitor IκB proteins^44^. Upon cellular stimulation, IκB is phosphorylated by IκB kinases (IKKs), and undergoes proteasomal degradation. The accumulation of phospho-Ser536 RelA in the nucleus traditionally represents as a marker for NF-κB signaling pathway activation^52^. Based on the current knowledge of NF-κB regulation, active NF-κB by such extracellular stimuli is translocated through the nuclear membrane in an “outside-in” manner to bind to DNA, and further either active or suppress gene expression^53^.

Our data indicated that SDF-1 induces RelA phosphorylation at Ser536 in EPCs through a novel non-canonical Erk/RSK/NF-κB signaling pathway, which in turn promotes basal nuclear RelA dissociation from its DNA promoter region and translocation from nucleus into cytoplasm. In agreement with our report, recent evidence indicated that a substantial amount of NF-κB (RelA) is present in the nucleus in several cell lines even in the absence of external cytokine stimuli^23,54–57^. We further showed a novel NF-κB regulatory mechanism, which mediates the translocation of active NF-κB in an “inside-out” manner to release it from DNA binding and to exert its transcriptional regulation. This non-canonical pathway of NF-κB activation/deactivation is a quite surprising finding and has never been delineated before.

Furthermore, this basal nuclear RelA regulates not only the expression of the CXCR4 receptor gene but also some genes of angiogenic cytokines, including IL-6, IL-8, and VEGF-A gene expressions through various NF-κB dimer combinations and different epigenetic mechanisms. A previous study has shown that GLP represses RelA target genes via recognition of SETD6-induced RelA methylation^23^. In contrast, we identified that in EPCs the inhibitory effect of GLP on CXCR4 transcription is exerted primarily via the interaction with basal nuclear RelA. Once RelA homodimer is phosphorylated by SDF-1 signaling and exported to the cytoplasm, GLP is also rapidly dissociated from chromatin, leading to attenuated H3K9 methylation. Subsequently, p50 homodimer binds to CXCR4 promoter and serves as a transcription activator by interaction with p300. Repression of IL-8 transcription by SDF-1 is due to dissociation of basal nuclear RelA-p50 heterodimer from IL-8 promoter, resulting in a repressive chromatin with H3K9 methylation by GLP. In addition, SDF-1 inhibits IL-6 and VEGF-A gene expression because the basal nuclear RelA homodimer is replaced by the p50 homodimer, leading to loss of p300 coactivator activity. Although mounting evidence indicates that angiogenic cytokines, such as IL-6, IL-8, and VEGF-A contribute to endothelial repair, there is no direct linkage between the restoration of SDF-1-inhibited cytokine expressions in EPCs by FGF23 versus the amelioration of AKI. Further researches are warranted in this aspect.

It is generally agreed that Klotho is necessary for FGF23 to activate FGFRs in Klotho-expressing cells. Our data show that Klotho does not participate in activation of FGF23/FGFR1 complex and its downstream Erk/RSK/RelA phosphorylation signaling. Another noteworthy finding is that loss of Klotho directly decreases RelA protein levels, resulting in upregulation of CXCR4 protein. In addition, this upregulation of CXCR4, which was induced by knockdown of Klotho, cannot be restored by FGF23 treatment. To sum up, although FGF23/FGFR1 signaling transduction is independent of Klotho, Klotho plays an important role in maintaining the level of RelA, a FGF23/FGFR1 downstream signaling molecule, which in turn indirectly regulates FGF23/FGFR1 signaling.

Several limitations also warrant mention. In our study, we only administered exogenous EPCs, and observed attenuation of AKI in the mouse IR-AKI model. Such AKI attenuation effect of EPCs were blocked by co-culture of the exogenous EPCs with exogenous SDF-1, but the AKI relieving effect was regained when exogenous FGF23 was added in vitro. Although engraftment of exogenous EPCs has indirectly proved that FGF23 mediates protection against IR-AKI through EPCs, we admit that we did not have any way to mark and track the endogenous late EPCs accurately in our FGF23-overexpressing IR-AKI mice; the contribution of FGF23-mediated endogenous EPCs awaits further elucidation.

In addition to modulate EPCs, FGF23 might also exert its multiple protective effects in IR-AKI simultaneously. As mentioned previously, neutrophils and renal tubular cells are potential candidate targets for FGF23. Furthermore, regulation of phosphate and 1,25 vitamin D hormone levels by FGF23 might also have an impact on IR-AKI progression. The different mechanisms underlying the protective effects of FGF23 against IR-AKI need further clarification.

In conclusion, our findings provide the experimental evidence FGF23 administration results in increased tubular cell regeneration, reduced cell death, and microvascular rarefaction in the IR-AKI mice kidneys. This novel finding, together with the roles of FGF23 contributing to the decrease of SDF-1-induced senescence of EPCs and suppression of SDF-1/CXCR4 via inhibition of a non-canonical Erk/RSK/NF-κB phosphorylation, may shed more insight for developing effective approaches to rescue AKI.

## Materials and methods

### Renal function

Renal function was assessed by measurements of the blood urea nitrogen (BUN-PIII dri-chem slide; FUJIFILM) and serum creatinine (CRE-PIII dri-chem slide; FUJIFILM) levels.

### Kidney injury score

Formalin-fixed kidney sections of 5-mm thickness were cut and stained with haematoxylin and eosin (H&E). Kidney injury score was graded for the number of necrotic cells using a semi-quantitative scale from 0 to 5, where 0 = none, 1 = <10%, 2 = 11 to 25%, 3 = 26 to 50%, 4 = 51 to 75% and 5 > 75%. The sections were evaluated in ten random high-power fields for each sample (*n* = 3). Kidney injury was scored in a random double-blind study by two investigators.

### TUNEL assay

Kidneys were fixed in 10% formalin, embedded in paraffin and sectioned. A terminal deoxynucleotidyltransferase-mediated dUTP-biotin nick end labeling (TUNEL) assay was performed using ApopTag® peroxidase in situ apoptosis detection kit (Millipore) following the manufacturer’s protocol. Nuclei were counterstained with hematoxylin. Mounted samples were analyzed by epifluorescent microscope (OLYMPUS BX51).

### Isolation of EPCs and cell culture

Total mononuclear cells (MNCs) were isolated from umbilical cord blood of healthy young human volunteers by Ficoll-Paque PLUS (GE Healthcare) density gradient centrifugation. MNCs (1 × 10^7^ cells) were plated in 10 ml endothelial cell growth medium-2 (EGM-2) MV (Lonza) with supplements containing VEGF, hFGF-B, hEGF, R3-IGF-1, hydrocortisone, ascorbic acid, 5% fetal bovine serum, and antibiotics on fibronectin-coated 10 cm cell culture plates at 37 °C in a 5% CO_2_ incubator. After 24 h of culture, non-adherent cells and debris were aspirated. Medium were changed every 3 days, and colonies of late EPCs appear after 2–3 weeks. The late EPCs exhibit a cobblestone endothelial-like morphology that is typical of mature endothelial cells at confluence. The late EPCs were characterized by the expression of CD31 (Abcam, #ab28364, 1:50), VE-cadherin (Cell signaling, #2158, 1:100), VEGF receptor 2 (Cell Signaling, #2479, 1:200), but negative for CD133 (Abcam, #ab19898, 1:200). Alexa Fluor 488-conjugated secondary antibody (Jackson lab, #111-545-003, 1:200) was used, and nuclei were counterstained with Hoechst 33342 (Invitrogen). Cell images were captured with an inverted fluorescence microscope (Axiovert 200M, Zeiss). Cells under passage 3 were used for late EPC studies. After serum starvation (0.5% FBS), the EPCs were treated with 100 ng/ml of recombinant SDF-1 (R&D) and 1 nM of recombinant human FGF23 (R&D) for the indicated times. EPCs were treated with 20 μM of PD98059 for Erk inhibitor (Cayman), 20 μM LY294002 for Akt inhibitor (Cayman), 10 μM FMK for RSK inhibitor (AdooQ Bioscience), 10 μM of Helenalin for RelA inhibitor (Cayman), 10 nM of Leptomycin B for nuclear export inhibitor (Cayman), 10 μM of PD173074 for FGFR inhibitor (Cayman), or DMSO as a negative control for 30 min prior to SDF-1 stimulation.

### Electrophoretic mobility shift assay (EMSA)

EPCs were pre-treated with FGF23 for 30 min followed by SDF-1 stimulation for 10 min. Treatment of NF-κB inhibitor (Helenalin) for 40 min was used as a positive control. Nuclear extracts were prepared using Nuclear/Cytosol Fractionation Kit (BioVision). EMSA was performed using the LightShift Chemiluminescent EMSA Kit (Thermo Scientific) according to the manufacturer’s instructions. The double-stranded oligonucleotide containing the NF-κB consensus sequence: 5′-CAGGGTCCCCTGGGCTTCCCAAGCCGCGCACCTCT-3′ presented in the promoter of CXCR4 at −130 to −164. Oligonucleotide probe was labeled with biotin at the both 5′ and 3′-end and incubated with nuclear extracts for 20 min at room temperature. For competition experiments, unlabeled CXCR4 probe was added at a 50-, 100- or 200-fold molar excess before the labeled probe. For the supershift assay, 2 µg of RelA (Santa Cruz, sc-8008X), p-RelA (Santa Cruz, sc-136548X) or p50 (Santa Cruz, sc-8414X) antibodies were pre-mixed with the nuclear extracts for 30 min on ice before adding the binding reaction mixture, further incubated for 20 min at room temperature. The resulting DNA–protein complexes were separated from the free oligonucleotides on 6% native polyacrylamide gel and transferred onto Immobilon-Ny+ Membrane (Millipore). The membrane was incubated with Streptavidin-HRP Conjugate, and then detected by chemiluminescence.

### Chromatin immunoprecipitation (ChIP) assay

ChIP assay was performed by SimpleChIP® Plus Enzymatic Chromatin IP Kit (Cell Signaling) according to the manufacturer’s instructions. EPCs were pre-treated with FGF23 for 30 min followed by SDF-1 stimulation for 15 min. Treatment of NF-κB inhibitor (Helenalin) for 40 min was used as a positive control. The cells were fixed with 1% formaldehyde, sonicated, and sheared into 150–900 bp of DNA fragments by micrococcal nuclease for 20 min at 37 °C. A portion of the cross-linked protein–DNA complexes were reserved as input DNA, and then the rest protein–DNA complexes were immunoprecipitated with RelA (Cell Signaling, #8242, 1:500), p-RelA (Santa Cruz, sc-136548X, 1:500), p50 (Santa Cruz, sc-8414X, 1:500), GLP (Abcam, ab41969, 1:250), H3K9me2 (Abcam, ab1220, 1:250), p300 (Abcam, ab14984, 1:250) or control (Cell Signaling, #2729, 1:500) antibodies. PCR analyses were carried out for 36 cycles with the following primer pairs: (5′-CGGACTCACTACCGACCAC-3′ & 5′-CGTCACTTTGCTACCTGCTG-3′) were used for CXCR4; (5′-GGTACATCCTCGACGGCATCT-3′ & 5′-GTGCCTCTTTGCTGCTTTCAC-3′) were used for IL-6; (5′-ACTGAGAGTGATTGAGAGTGGAC-3′ & 5′-AACCCTCTGCACCCAGTTTTC-3′) were used for IL-8; and (5′-CTACCTCCACCATGCCAAGT-3′ & 5′-GCAGTAGCTGCGCTGATAGA-3′) are used for VEGF-A.

### Immunoprecipitation and western blot analysis

Mouse kidneys and EPCs were homogenized with RIPA buffer (50 mM Tris, pH 7.4, 150 mM NaCl, 1% Nonidet P-40, 0.25% Na-deoxycholate, 0.1% SDS, 1 mM PMSF, 1 mM NaF, 1 mM Na3VO4, phosphatase inhibitor cocktail (Roche) and protease inhibitor cocktail (Roche)). Nuclear extracts were prepared using Nuclear/Cytosol Fractionation Kit (BioVision). For immunoprecipitation experiments, cell lysates were incubated with RelA (Cell Signaling, #8242, 1:100) antibody overnight at 4 °C and immunoprecipitated with Protein G beads (Millipore). Bound complexes were resolved on SDS-PAGE gels and analyzed by Western blotting. Western blot analysis was performed at least in triplicate. The following commercial primary antibodies were used for immunoprecipitation and/or western blots: PCNA (Cell Signaling, #13110, 1:2000), p-Akt (Cell Signaling, #4060, 1:2000), E-cadherin (Cell Signaling, #3195, 1:2000), CXCR4 (Abcam, ab2074, 1:1000), Erk (Cell Signaling, #9102, 1:10000), p-Erk (Cell Signaling, #9101, 1:10000), RSK (Cell Signaling, #9355, 1:5000), p-RSK (S380) (Cell Signaling, #11989, 1:2000), p-RSK (T359) (Cell Signaling, #8753, 1:1000), p-RSK (T573) (Cell Signaling, #9346, 1:1000), RelA (Cell Signaling, #8242, 1:5000), p-RelA (Cell Signaling, #3033, 1: 1000), GLP (Bethyl, A301-642A, 1:2000), Klotho (Thermo, PA5-21078, 1:2000), IKKα (Cell Signaling, #11930, 1:2000), IKKβ (Cell Signaling, #8943, 1:2000), p-IKKα/β (Cell Signaling, #2697, 1:1000), IκB (Cell Signaling, #4814, 1:5000), p-IκB (Cell Signaling, #2859, 1:1000), Histone H3 (Cell Signaling, #4499, 1:5000), β-actin (Santa Cruz, sc-69879, 1:10,000), and GAPDH (Millipore, MAB374, 1:10,000). Incubation with anti-mouse IgG HRP (Jackson Lab, 115-025-166, 1:50,000) and anti-rabbit IgG HRP (Jackson Lab, 111-035-144, 1:50,000), followed by ECL was used for detection. Ponceau S staining was served as the mouse kidney loading control. The western blots were quantified using ImageJ (http://rsbweb.nih.gov/ij/).

### Immunohistochemistry and immunofluorescence

For immunohistochemistry, kidney samples were fixed in 10% Formalin, embedded in paraffin, and sectioned. Sections were heated in DAKO Target Retrieval Solution (pH 6.0) for antigen retrieval, blocked in 5% BSA for 1 h at room temperature, and incubated with primary antibody at 4 °C overnight. The following commercial primary antibodies were used for immunohistochemistry: PCNA (Cell Signaling, #13110, 1:2000), p-Akt (Cell Signaling, #4060, 1:50), or CD31 (Cell Signaling, #77699, 1:100). Then, samples were incubated with SignalStain® Boost IHC Detection Reagent (Cell Signaling, #8114) and exposed to DAB for colorimetric detection. Nuclei were counterstained with hematoxylin. Images were captured by epifluorescent microscope (OLYMPUS BX51) and analyzed using ImageJ (http://rsbweb.nih.gov/ij/). The percentage of positive cells was calculated in ten random high-power fields (×400) for each triplicate sample.

For immunocytochemistry, serum-starved EPCs were grown on 4-chamber slides. After treatment of SDF-1 and/or FGF23, cells were fixed with cold 4% paraformaldehyde (PFA) for 15 min, and then incubated in NH4Cl/PBS (50 mM) for 10 min. Cells were permeabilized with 0.25% Triton X-100, blocked in 5% BSA for 30 min at room temperature, and incubated with primary antibodies at 4 °C overnight. The following commercial primary antibodies were used for immunocytochemistry: Erk (Cell Signaling, #9102, 1:50), p-Erk (Cell Signaling, #9101, 1:500), RSK (Cell Signaling, #9355, 1:25), p-RSK (Cell Signaling, #11989, 1:800), RelA (Cell Signaling, #8242, 1:400), and p-RelA (Cell Signaling, #3033, 1:100). Alexa Fluor 488-conjugated secondary antibody (Jackson lab, #111-545-003, 1:200) was used, and nuclei were counterstained with Hoechst 33342 (Invitrogen). Mounted cells were analyzed by confocal laser scanning microscopy using a Zeiss LSM Meta 510.

### RT-PCR and quantitative real-time PCR

Total RNA was extracted from EPCs using TRIzol reagent (Invitrogen). Reverse transcription reactions were performed at 42 °C using BioScript cDNA synthesis kit (Bioline). RT-PCR analyses were carried out using MyTaq HS Mix (Bioline) with the following primer pairs: (5′-GAAGTTCAAATGCCCTTCCA-3′ & 5′-TCGATGTGCTTTAGCCACTG-3′) were used for FGFR1; (5′-CAGGGGTCTCCGAGTATGAA-3′ & 5′-TCTCGGAGGTTGCCTTTAGA-3′) were used for FGFR2; (5′-ACTGTCTGGGTCAAGGATGG-3′ & 5′-GTTCTTCAGCCAGGAGATGG-3′) were used for FGFR3; and (5′-CAAAGACAACGCCTCTGACA-3′ & 5′-TGGATACACTTCCGGGACTC-3′) were used for FGFR4. Quantitative real-time PCR (q-PCR) analyses were performed using SYBR Green PCR master mix (Roche) with the following primer pairs: (5′-CTGCCCAGAAGGGAAGCGTGATGA-3′ & 5′-TGACGGACAAGTACAGGCTGC-3′) were used for CXCR4; (5′-GGTACATCCTCGACGGCATCT-3′ & 5′-GTGCCTCTTTGCTGCTTTCAC-3′) were used for IL-6; (5′-ACTGAGAGTGATTGAGAGTGGAC-3′ & 5′-AACCCTCTGCACCCAGTTTTC-3′) were used for IL-8; (5′-CTACCTCCACCATGCCAAGT-3′ & 5′-GCAGTAGCTGCGCTGATAGA-3′) were used for VEGF-A; and (5′-TGCCGACAGGATGCGAAG-3′ & 5′-CGCTCAGGAGGAGCAATGA-3′) were used for actin. The relative mRNA expression levels were calculated according to the ΔΔCt method and normalized to actin.

### Electroporation-mediated gene delivery

For the Klotho knockdown assay, pGFP-C-shLenti vectors containing shRNA targeting human Klotho (TL303679A) or non-targeting scrambled shRNA cassette (TR30023) were purchased from Origene. For the RelA knockdown assay, SignalSilence NF-κB p65 siRNA II (#6534) and SignalSilence Control siRNA (#6210) were purchased from Cell Signaling. For RelA overexpression assay, pcDNA3.1^+^/C-(K)DYK vectors containing human WT (NM_021975), S536A, or S536E RelA were purchased from GenScript. EPCs were nucleofected with Klotho shRNA, RelA siRNA, WT RelA, S536A RelA, or S536E RelA using 4D-Nucleofector™ X Kit (Lonza), program CA-167.

### Proliferation assay

The proliferation of late EPCs was determined by Cell Counting Kit-8 (CCK-8) assay (Dojindo Laboratories). In brief, ~1000 cells of EPCs were plated in triplicate in 96-well plates and cultured in EGM-2 MV medium with 100 ng/ml SDF-1 and/or 1 nM FGF23. Cell proliferation was measured every 24 h for up to 5 days. The absorbance was measured at 450 nm using a spectrophotometer.

### Apoptosis assay

Apoptosis of EPCs was induced by serum starvation. In brief, ~2.5 × 10^4^ cells of EPCs were seeded onto in triplicate in 96-well plates. After 24 h of incubation, culture medium was removed and replaced with EBM-2 medium without any supplement in the present of 100 ng/ml SDF-1 and/or 1 nM FGF23. After 48 h of serum deprivation, Cell Counting Kit-8 (CCK-8) assay (Dojindo Laboratories) was used to determine cell viability.

### Migration assay

The migratory function of EPCs was evaluated by a modified Boyden chamber (Transwell, Coster) assay. In brief, SDF-1 was diluted to 100 ng/ml in EGM-2 MV medium with 10% fetal bovine serum, and 600 μl of the final dilution was placed in the lower compartment of a Boyden chamber. Serum-starved EPCs were treated with 100 ng/ml SDF-1 and/or 1 nM FGF23 for 24 h, and then 3 × 10^4^ cells were suspended in 100 μl of EGM-2 MV medium supplemented with 0.5% fetal bovine serum and reseeded in the upper compartment. After incubation for 3 h at 37 °C, the filters were stained with Hoechst 33342 (Invitrogen) and migrated EPCs were counted in six random fields (100X) for each triplicate sample.

### In vitro endothelial cell-specific angiogenesis

The angiogenic effect of SDF-1 and/or FGF23 on EPCs was assessed using a previously described co-culture assay^58^. Human fetal lung fibroblasts (MRC-5) were seeded on 4-chamber slides and allowed to grow to confluence. Suspensions of EPCs (5000 cells/well) were placed on the monolayers for 6 days in EGM-2 MV medium with 100 ng/ml SDF-1 and/or 1 nM FGF23. At the end of the assay, endothelial cells were stained with CD31 antibody (Abcam, #ab28364, 1:200). Then, samples were incubated with SignalStain® Boost IHC Detection Reagent (Cell Signaling, #8114) and exposed to DAB for colorimetric detection. Images were captured by epifluorescent microscope (OLYMPUS BX51) and analyzed using ImageJ (http://rsbweb.nih.gov/ij/). Color images were binarized and threshold-adjusted to obtain optimal contrast of tubules against the background. The endothelial cell area (CD31 positive cells) was counted in ten random high-power fields (400X) for each triplicate sample.

### Senescence-associated β-galactosidase assay

Serum-starved EPCs were treated with 100 ng/ml SDF-1 and/or 1 nM FGF23 for 5 days. Senescence-associated beta-galactosidase (SA-β-gal) activity was measured by Senescence detection Kit (Merck Millipore). Cell nuclei were counterstained with Hoechst 33342 (Invitrogen). Total and β-gal positive cells were counted in ten random high-power fields (×400) for each triplicate sample.

### Enzyme-linked immunosorbent assays (ELISA)

Circulating mouse intact form FGF23 (iFGF23) were measured using standard enzyme-linked immunosorbent assay (ELISA) kits (Immutopics) according to the manufacturer’s instructions. To assess cytokine secretion, EPCs were pre-treated with FGF23 for 30 min followed by SDF-1 stimulation for 48 h. VEGF-A, IL-6, and IL-8 were measured in culture supernatants using standard ELISA kits (R&D) according to the manufacturer’s instructions.

### IR-AKI mice models

Male C57BL/6 and NOD-SCID mice (NOD.CB17-Prkdcscid/NcrCrlBltw) (20 ± 2 g body weight or 8–9 weeks of age) were briefly anesthetized with an intraperitoneal injection of anesthesia cocktails (35 mg/kg zoletil plus 5 mg/kg xylazine). Through a midline incision, right nephrectomy was performed and the left renal artery was clamped for 30 min with a microaneurysm clamp. The body temperature of the mice was maintained at 33 °C by a heating pad. Animal was excluded if quantification of BUN and Cre was not elevated after IR-AKI.

### Time-course analysis of IR-AKI

C57BL/6 mice were randomly assigned to 2 groups as follows: (1) sham group: sham-operated mice were subjected to the same surgical procedure except that the left renal pedicles were not clamped; and (2) IR-AKI group: right nephrectomy first and followed by left renal artery clamping. Mice were sacrificed (6 animals in per group) at 1, 3, 5, 8, 12, 24, and 48 h after surgery, respectively.

### Exogenous FGF23 administration in IR-AKI mice

C57BL/6 mice were randomly assigned to 4 groups as follows: (1) sham + vehicle group; (2) sham + FGF23 group; (3) IR-AKI + vehicle group; and (4) IR-AKI + FGF23 group. Mice in FGF23 groups received intravenous injection of recombinant mouse FGF23 (6His-tagged Tyr25-Val251 [Arg179Gln]) proteins (R&D) as previously described^29^. Briefly, 40 μg/kg FGF23 dissolved in 200 μl PBS were injected into the tail vein at 30 min before surgery, and then twice daily with 8 h between injections for 2 consecutive days. Mice in vehicle groups received the same injection schedule using 200 μl PBS alone as negative control. Mice were sacrificed (9 animals in per group) at 48 h after IR-AKI.

### In vivo overexpression of FGF23 in IR-AKI mice

C57BL/6 mice were randomly assigned to 2 groups as follows: (1) IR-AKI + pcDNA3.1 group and (2) IR-AKI + pFGF23 group. For FGF23 overexpression assay, pcDNA3.1 + /C-DYK vectors containing mutant cleavage-resistant form of mouse FGF23 were purchased from GenScript. In vivo plasmid-based gene delivery was carried out using *Trans*IT®-EE Hydrodynamic Delivery Solution (Mirus Bio)^59^. Briefly, FGF23 expression plasmid (pFGF23) or empty vector (pcDNA3.1) were diluted with Mirus *Trans*IT®-EE solution at 10 μg/ml (in volume ~10% of mouse body weight) and injected through the tail vein at 24 h before surgery. Mice were sacrificed (9 animals in per group) at 48 h after IR-AKI.

### Administration of EPCs in IR-AKI SCID mice

To prevent immunorejection of human EPCs, experiments were performed in NOD-SCID mice. Mice were randomly assigned to 6 groups as follows: (1) sham + vehicle group; (2) IR-AKI + vehicle group; (3) IR-AKI + EPCs group; (4) IR-AKI + EPCs (SDF-1-treated) group; (5) IR-AKI + EPCs (SDF-1/FGF23-treated) group; and (6) IR-AKI + EPCs (FGF23-treated) group. EPCs were ex vivo pre-treated with SDF-1 and/or FGF23 for 24 h before EPC injection. Mice in EPCs groups received EPCs (1 × 10^6^ cells/mouse) intravenously after IR-AKI surgery immediately. Mice in vehicle groups received the same injection schedule using PBS as negative control. Mice were sacrificed (6 animals in per group) at 48 h after IR-AKI.

### Statistical analysis

Data were expressed as mean ± standard deviation (SD) or standard error of the mean (SEM) from at least three independent experiments. Comparisons of two groups were analyzed by Student’s *t*-test. A probability *p*-value less than 0.05 was considered statistically significant. Asterisks represent different levels of statistical power: *p < 0.05, **p < 0.01, ***p < 0.001.

## Supplementary information

Supplementary data

Supplementary Fig. 1

Supplementary Fig. 2

Supplementary Fig. 3

Supplementary Fig. 4

Supplementary Fig. 5-1

Supplementary Fig. 5-2

Supplementary Fig. 6

Supplementary Fig. 7

Supplementary Fig. 8

Supplementary Fig. 9

## References

[1] Leaf DE, Wolf M, Stern L (2010). Elevated FGF-23 in a patient with rhabdomyolysis-induced acute kidney injury. Nephrol., Dial. Transplant..

[2] Zhang M (2011). FGF-23 and PTH levels in patients with acute kidney injury: a cross-sectional case series study. Ann. Intensive Care.

[3] Christov M (2013). Plasma FGF23 levels increase rapidly after acute kidney injury. Kidney Int..

[4] Leaf DE (2016). Fibroblast growth factor 23 levels are elevated and associated with severe acute kidney injury and death following cardiac surgery. Kidney Int..

[5] Ali FN, Hassinger A, Price H, Langman CB (2013). Preoperative plasma FGF23 levels predict acute kidney injury in children: results of a pilot study. Pediatr. Nephrol. (Berl., Ger.).

[6] Leaf DE (2013). Dysregulated mineral metabolism in patients with acute kidney injury and risk of adverse outcomes. Clin. Endocrinol. (Oxf.).

[7] Brown JR (2014). Fibroblast growth factor-23 and the long-term risk of hospital-associated AKI among community-dwelling older individuals. Clin. J. Am. Soc. Nephrology..

[8] Leaf DE (2012). FGF-23 levels in patients with AKI and risk of adverse outcomes. Clin. J. Am. Soc. Nephrol..

[9] Hanudel MR (2016). Effects of acute kidney injury and chronic hypoxemia on fibroblast growth factor 23 levels in pediatric cardiac surgery patients. Pediatr. Nephrol. (Berl., Ger.).

[10] Leaf D. E. et al. Fibroblast growth factor 23 levels associate with AKI and death in critical illness. *J. Am. Soc. Nephrol.* **28**,1877–1885 (2016). 10.1681/ASN.2016080836 PMC5461795 28028134

[11] Bonventre JV, Yang L (2011). Cellular pathophysiology of ischemic acute kidney injury. J. Clin. Invest..

[12] Cantaluppi V (2012). [New mechanisms and recent insights in the pathogenesis of acute kidney injury (AKI)]. G Ital. Nefrol..

[13] Molitoris BA, Sutton TA (2004). Endothelial injury and dysfunction: role in the extension phase of acute renal failure. Kidney Int..

[14] Pearson JD (2010). Endothelial progenitor cells–an evolving story. Microvasc. Res.

[15] Patschan D, Patschan S, Muller GA (2011). Endothelial progenitor cells in acute ischemic kidney injury: strategies for increasing the cells’ renoprotective competence. Int. J. Nephrol..

[16] Wu VC (2013). In acute kidney injury, indoxyl sulfate impairs human endothelial progenitor cells: modulation by statin. Angiogenesis.

[17] Liang CJ (2015). Endothelial progenitor cells derived from Wharton’s jelly of human umbilical cord attenuate ischemic acute kidney injury by increasing vascularization and decreasing apoptosis, inflammation, and fibrosis. Cell Transplant..

[18] Ganju RK (1998). The alpha-chemokine, stromal cell-derived factor-1alpha, binds to the transmembrane G-protein-coupled CXCR-4 receptor and activates multiple signal transduction pathways. J. Biol. Chem..

[19] Lai P (2008). Upregulation of stromal cell-derived factor 1 (SDF-1) expression in microvasculature endothelial cells in retinal ischemia-reperfusion injury. Graefes Arch. Clin. Exp. Ophthalmol..

[20] Vagima Y (2011). Pathways implicated in stem cell migration: the SDF-1/CXCR4 axis. Methods Mol. Biol. (Clifton, NJ).

[21] Liu N, Tian J, Cheng J, Zhang J (2013). Migration of CXCR4 gene-modified bone marrow-derived mesenchymal stem cells to the acute injured kidney. J. Cell Biochem.

[22] Lyss G, Knorre A, Schmidt TJ, Pahl HL, Merfort I (1998). The anti-inflammatory sesquiterpene lactone helenalin inhibits the transcription factor NF-kappaB by directly targeting p65. J. Biol. Chem..

[23] Levy D (2011). Lysine methylation of the NF-kappaB subunit RelA by SETD6 couples activity of the histone methyltransferase GLP at chromatin to tonic repression of NF-kappaB signaling. Nat. Immunol..

[24] Barski A (2007). High-resolution profiling of histone methylations in the human genome. Cell.

[25] Yang Z (2010). Paracrine factors secreted by endothelial progenitor cells prevent oxidative stress-induced apoptosis of mature endothelial cells. Atherosclerosis.

[26] Di Santo S, Seiler S, Fuchs AL, Staudigl J, Widmer HR (2014). The secretome of endothelial progenitor cells promotes brain endothelial cell activity through PI3-kinase and MAP-kinase. PLoS ONE.

[27] He T, Peterson TE, Katusic ZS (2005). Paracrine mitogenic effect of human endothelial progenitor cells: role of interleukin-8. Am. J. Physiol. Heart Circ. Physiol..

[28] Shalhoub V (2012). FGF23 neutralization improves chronic kidney disease-associated hyperparathyroidism yet increases mortality. J. Clin. Invest..

[29] Faul C (2011). FGF23 induces left ventricular hypertrophy. J. Clin. Invest..

[30] de Jong M. A. et al. Fibroblast growth factor 23 modifies the pharmacological effects of angiotensin receptor blockade in experimental renal fibrosis. *Nephrol. Dial. Transplant.* **32**, 73–80 (2017). 10.1093/ndt/gfw105 27220755

[31] Medici D (2008). FGF-23-Klotho signaling stimulates proliferation and prevents vitamin D-induced apoptosis. J. Cell Biol..

[32] Linas SL, Shanley PF, Whittenburg D, Berger E, Repine JE (1988). Neutrophils accentuate ischemia-reperfusion injury in isolated perfused rat kidneys. Am. J. Physiol..

[33] Linas SL, Whittenburg D, Parsons PE, Repine JE (1995). Ischemia increases neutrophil retention and worsens acute renal failure: role of oxygen metabolites and ICAM 1. Kidney Int..

[34] Kato N (2009). The E-selectin ligand basigin/CD147 is responsible for neutrophil recruitment in renal ischemia/reperfusion. J. Am. Soc. Nephrol..

[35] Kelly KJ (1996). Intercellular adhesion molecule-1-deficient mice are protected against ischemic renal injury. J. Clin. Invest..

[36] Rossaint J. et al. FGF23 signaling impairs neutrophil recruitment and host defense during CKD. *J. Clin. Invest.* 126, 962–974 (2016). 10.1172/JCI83470 PMC4767336 26878171

[37] Shiba Y (2009). Bone marrow CXCR4 induction by cultivation enhances therapeutic angiogenesis. Cardiovasc. Res..

[38] Chiang KH (2015). Statins, HMG-CoA reductase inhibitors, improve neovascularization by increasing the expression density of CXCR4 in endothelial progenitor cells. PLoS ONE.

[39] Sradnick J (2016). Extrarenal progenitor cells do not contribute to renal endothelial repair. J. Am. Soc. Nephrol..

[40] Burger D (2015). Human endothelial colony-forming cells protect against acute kidney injury: role of exosomes. Am. J. Pathol..

[41] Briasoulis A, Tousoulis D, Antoniades C, Papageorgiou N, Stefanadis C (2011). The role of endothelial progenitor cells in vascular repair after arterial injury and atherosclerotic plaque development. Cardiovasc. Ther..

[42] Fan H (2014). Endothelial progenitor cells and a stromal cell-derived factor-1alpha analogue synergistically improve survival in sepsis. Am. J. Respir. Crit. Care Med..

[43] Lin Y, Weisdorf DJ, Solovey A, Hebbel RP (2000). Origins of circulating endothelial cells and endothelial outgrowth from blood. J. Clin. Invest..

[44] Baldwin AS (1996). The NF-kappa B and I kappa B proteins: new discoveries and insights. Annu. Rev. Immunol..

[45] Ghosh S, May MJ, Kopp EB, NF-kappa B (1998). and Rel proteins: evolutionarily conserved mediators of immune responses. Annu. Rev. Immunol..

[46] Karin M, Lin A (2002). NF-kappaB at the crossroads of life and death. Nat. Immunol..

[47] Lenardo MJ, Baltimore D (1989). NF-kappa B: a pleiotropic mediator of inducible and tissue-specific gene control. Cell.

[48] Lin A, Karin M (2003). NF-kappaB in cancer: a marked target. Semin. Cancer Biol..

[49] Thanos D, Maniatis T (1995). NF-kappa B: a lesson in family values. Cell.

[50] Verma IM, Stevenson JK, Schwarz EM, Van Antwerp D, Miyamoto S (1995). Rel/NF-kappa B/I kappa B family: intimate tales of association and dissociation. Genes Dev..

[51] Hoffmann A, Natoli G, Ghosh G (2006). Transcriptional regulation via the NF-kappaB signaling module. Oncogene.

[52] Perkins ND (2006). Post-translational modifications regulating the activity and function of the nuclear factor kappa B pathway. Oncogene.

[53] Liou HC, Baltimore D (1993). Regulation of the NF-kappa B/rel transcription factor and I kappa B inhibitor system. Curr. Opin. Cell Biol..

[54] Frankenberger M (1994). Constitutive nuclear NF-kappa B in cells of the monocyte lineage. Biochem J..

[55] Delfino F, Walker WH (1998). Stage-specific nuclear expression of NF-kappaB in mammalian testis. Mol. Endocrinol..

[56] Naskar D (2014). Wnt5a-Rac1-NF-kappaB homeostatic circuitry sustains innate immune functions in macrophages. J. Immunol..

[57] Liu J (2006). NF-kappaB is required for UV-induced JNK activation via induction of PKCdelta. Mol. Cell.

[58] Sieveking DP, Buckle A, Celermajer DS, Ng MK (2008). Strikingly different angiogenic properties of endothelial progenitor cell subpopulations: insights from a novel human angiogenesis assay. J. Am. Coll. Cardiol..

[59] Xie J, Yoon J, An SW, Kuro-o M, Huang CL (2015). Soluble Klotho protects against uremic cardiomyopathy independently of fibroblast growth factor 23 and phosphate. J. Am. Soc. Nephrol..

