# Supplementary material for: FGF23 ameliorates ischemia-reperfusion induced acute kidney injury via modulation of endothelial progenitor cells: targeting SDF-1/CXCR4 signaling
Source: Cell Death Dis. 2021 Apr 17;12(5):409. doi: 10.1038/s41419-021-03693-w (PMC8053200; doi:10.1038/s41419-021-03693-w)
Supplement: Supplementary file 1 — Supplementary data [file 41419_2021_3693_MOESM1_ESM.doc]

**Supplementary figure legends**

**Supplementary Fig. 1** Characterization of ischemia-reperfusion injury (IRI) in mice. **a, b** Serum blood urea nitrogen (BUN) (**a**) and creatinine (CRE) (**b**) were measured at the indicated time points following IRI. Bars on graphs are ± SEM (n=6). **c** Plasma levels of iFGF23 were determined using ELISA kit at the indicated time points following IRI. Bars on graph are ±SEM (n=3). ****p* < 0.001 indicates the earliest time point at which a significant difference between IR-AKI and control mice by t test.

**Supplementary Fig. 2** FGF23 enhances renal regenerative and anti-apoptosis activity after 48 hours of reperfusion. **a** Kidney were harvested after 48 hours of reperfusion and immunoblotted with the indicated antibodies. Ponceau S staining was used as a protein loading control. The numbers under the gel lanes represent the relative protein level. **b** Immunohistochemical (IHC) demonstration of p-Akt in kidney tissues. **c** IHC demonstration of PCNA (Proliferating Cell Nuclear Antigen) in kidney tissues. **d** Kidneys were subjected to TUNEL staining. Representative results of Western blot analysis were obtained from two independent mice from each group. The percentages of positive cells were counted in ten random fields for each sample (n=3). Scale bars, 20 μm. Bars on graphs are ± SEM. ****p* < 0.001 by t test.

**Supplementary Fig. 3** Characterization of human umbilical cord blood-derived late EPCs. Immunofluorescence staining was performed with indicated antibodies in EPCs. Late EPCs expressed endothelial markers, CD31, VE-cadherin, and VEGFR-2, but without hematopoietic stem cell marker CD133. Negative control was incubated with secondary antibody only. Nuclei were counterstained with Hoechst 33342. Cells were visualized by an inverted fluorescence microscope (HPF, 400x). Scale bars, 60 μm.

**Supplementary Fig. 4** SDF-1 augments CXCR4 expression via the non-canonical NF-κB signaling pathway in EPCs. **a-c** EPCs were harvested at the indicated time points after SDF-1 treatment. **d** EPCs were pre-treated with FGF23 for 30 minutes followed by SDF-1 stimulation for 10 minutes or 24 hours. Protein lysates were analyzed by immunoblot using indicated antibodies. GAPDH was used as a protein loading control. The numbers under the gel lanes represent the relative protein level.

**Supplementary Fig. 5** SDF-1 induces the export of basal nuclear NF-κB (RelA) from nucleus to cytoplasm. **a** EPCs were pre-treated with FGF23 for 30 minutes followed by SDF-1 stimulation for 10 minutes. Treatment of NF-κB inhibitor (Helenalin) for 40 minutes was used as a positive control. **b** EPCs were pre-treated with FGF23 for 30 minutes followed by SDF-1 stimulation for 10 minutes. Protein lysates were analyzed by immunoblot using indicated antibodies. Histone H3 was used as a nuclear protein loading control. The numbers under the gel lanes represent the relative protein level. **c** EPCs were pre-treated with the inhibitor of nuclear export, leptomycin B (LMB) for 30 minutes followed by SDF-1 stimulation for 10 minutes. Immunofluorescence staining was performed with indicated antibodies in EPCs (**a, c**). Nuclei were counterstained with Hoechst 33342. Cells were visualized by confocal laser scanning microscopy (HPF, 400x).

**Supplementary Fig. 6** SDF-1 induces CXCR4 expression through abolishment of GLP-mediated epigenetic gene repression. **a** EPCs were treated with GLP inhibitor (UNC0224) for 24 hours. Protein lysates were analyzed by immunoblot using CXCR4 antibody. β-actin was used as a protein loading control. **b** Lysates from untreated EPCs were subjected to co-immunoprecipitation with RelA antibody and the precipitates were analyzed by Western blotting using GLP or RelA antibodies. **c-e** EPCs were pre-treated with FGF23 for 30 minutes followed by SDF-1 stimulation for 15 minutes (**c, e**) or 1 hour (**d**). For ChIP assays, cross-linked chromatin were immunoprecipitated with GLP (**c**), H3K9me2 (**d**), or p300 (**e**) antibodies and subjected to PCR with CXCR4 specific primers. Treatment with IgG antibody was used as a negative control. PCR product of unprecipitated chromosomal DNA was used as an input control.

**Supplementary Fig. 7** RelA conducts as a transcriptional activator of angiogenic cytokines in EPCs. **a, b, e-g** EPCs were pre-treated with FGF23 for 30 minutes followed by SDF-1 stimulation for 15 minutes (**a, b, e, g**) or 1 hour (**f**). For ChIP assays, cross-linked chromatin were immunoprecipitated with RelA (**a**), p50 (**b**), GLP (**e**), H3K9me3 (**f**), or p300 (**g**) antibodies and subjected to PCR with indicated specific primers. Treatment with IgG antibody was used as a negative control. PCR product of unprecipitated chromosomal DNA was used as an input control. **c** EPCs were pre-treated with FGF23 for 30 minutes followed by SDF-1 stimulation for 15 minutes. VEGF-A, IL-6, or IL-8 mRNA expression levels were shown by q-PCR, and normalized to actin. Bars on graphs are ± SD. ****p* < 0.001 by t test, n=3. **d** EPCs were pre-treated with FGF23 for 30 minutes followed by SDF-1 stimulation for 48 hours. Cell culture supernatants were analyzed by ELISA for VEGF-A, IL-6, or IL-8 levels. Bars on graphs are ± SD. **p* < 0.05, ***p* < 0.01, ****p* < 0.001 by t test, n=3.

**Supplementary Fig. 8** Epigenetic changes of CXCR4, VEGF-A, IL-6, and IL-8 genes are regulated by basal nuclear NF-κB in EPCs. **a** Dissociation of RelA homodimer from CXCR4 promoter attenuates DNA methylation, leading to gene activation. **b** Dissociation of RelA homodimer from VEGF-A and IL-6 promoters attenuates DNA acetylation, leading to gene repression. **c** Dissociation of RelA-p50 heterodimer from IL-8 promoter augments DNA methylation, leading to gene repression.

**Supplementary Fig. 9** Schematic representation of the cross-talk between SDF-1 and FGF23 signaling pathways in EPCs. SDF-1 induces its receptor CXCR4 upregulation through Erk/RSK/RelA signaling pathway, creating a positive feedback loop that promotes EPC migration, senescence, and angiogenesis. However, FGF23 suppresses SDF-1 signaling pathway via inhibition of Erk phosphorylation in a Klotho-independent manner. FGF23 attenuates migration and senescence of EPCs induced by SDF-1, but not angiogenesis.
