# Supplementary figures and images for: FGF23 ameliorates ischemia-reperfusion induced acute kidney injury via modulation of endothelial progenitor cells: targeting SDF-1/CXCR4 signaling
Source: Cell Death Dis. 2021 Apr 17;12(5):409. doi: 10.1038/s41419-021-03693-w (PMC8053200; doi:10.1038/s41419-021-03693-w)

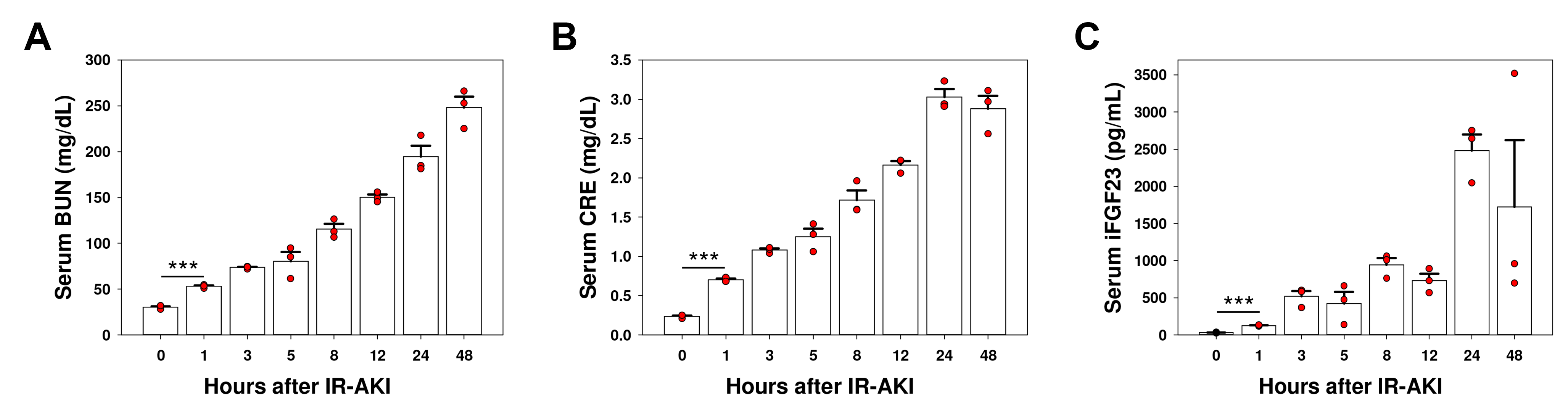

Supplement: Supplementary file 2 — Supplementary Fig. 1 [file 41419_2021_3693_MOESM2_ESM.tif]

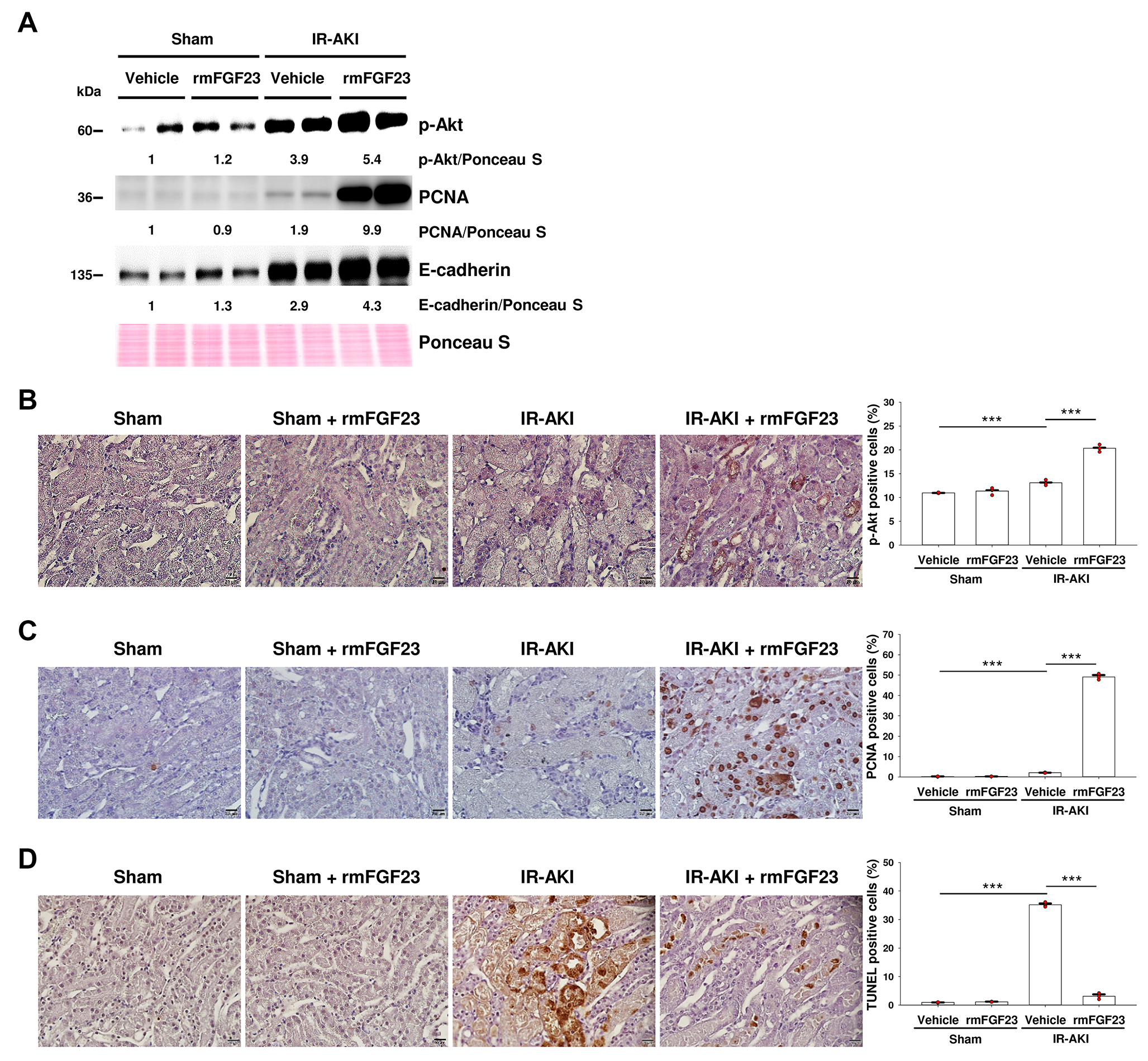

Supplement: Supplementary file 3 — Supplementary Fig. 2 [file 41419_2021_3693_MOESM3_ESM.tif]

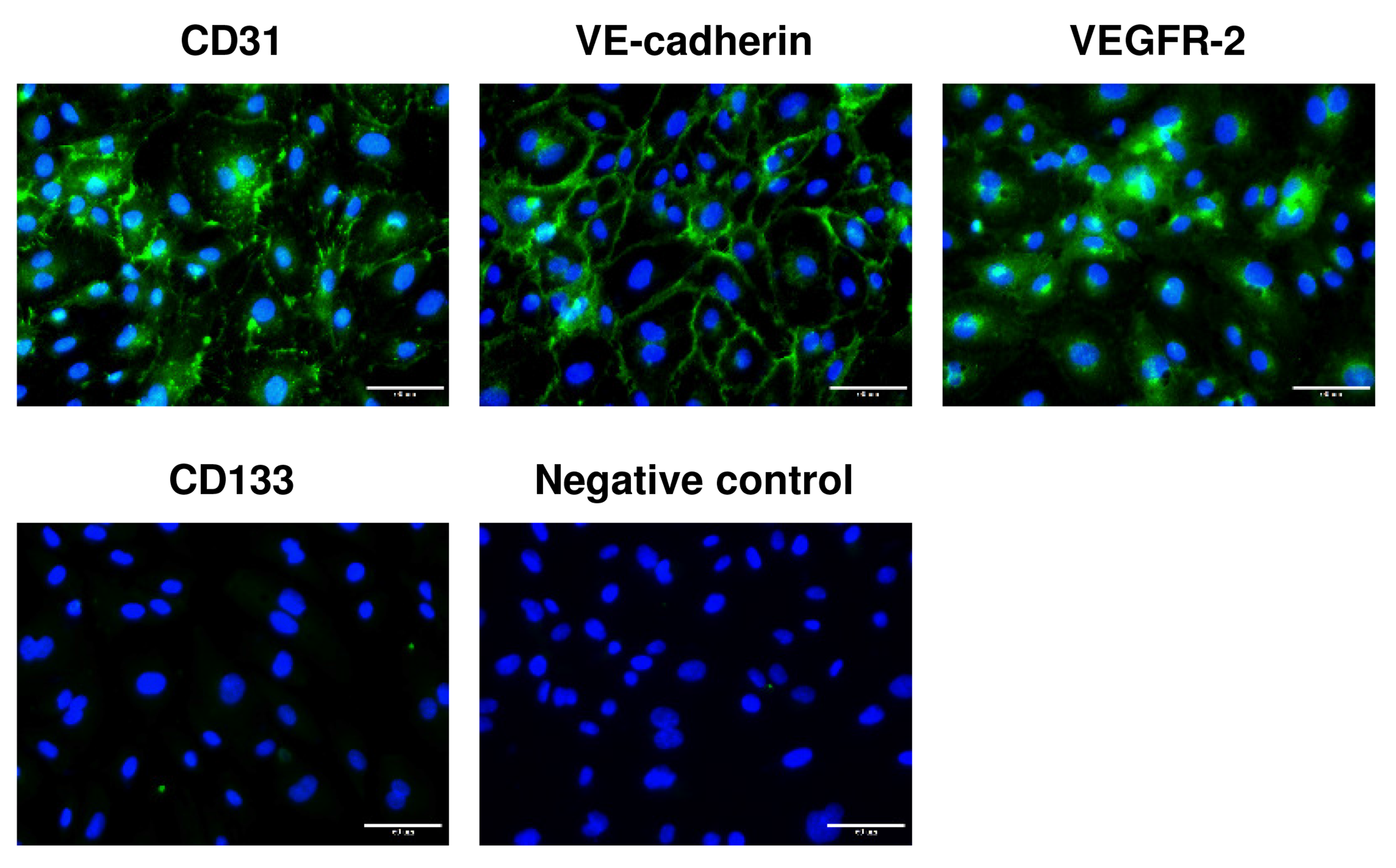

Supplement: Supplementary file 4 — Supplementary Fig. 3 [file 41419_2021_3693_MOESM4_ESM.tif]

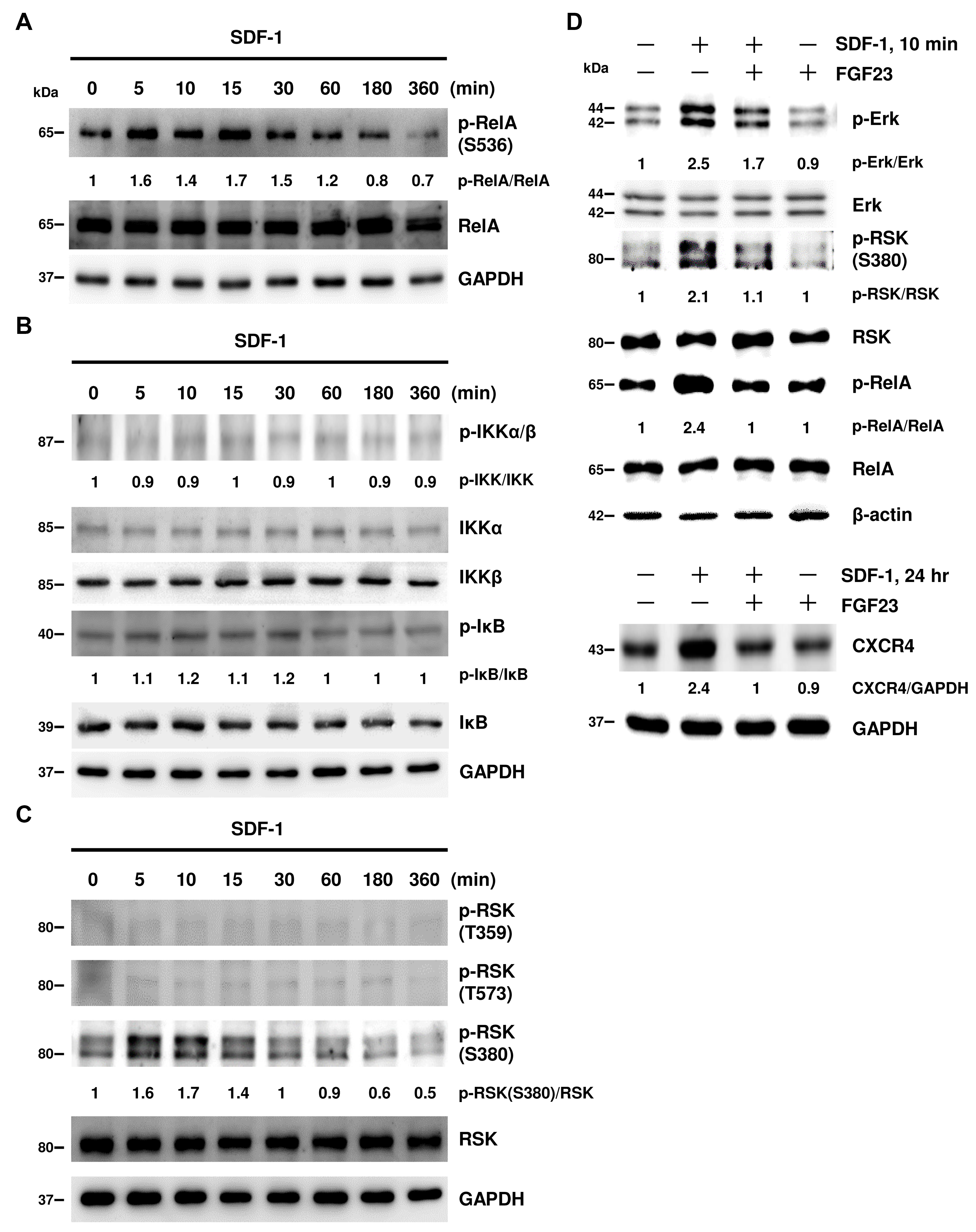

Supplement: Supplementary file 5 — Supplementary Fig. 4 [file 41419_2021_3693_MOESM5_ESM.tif]

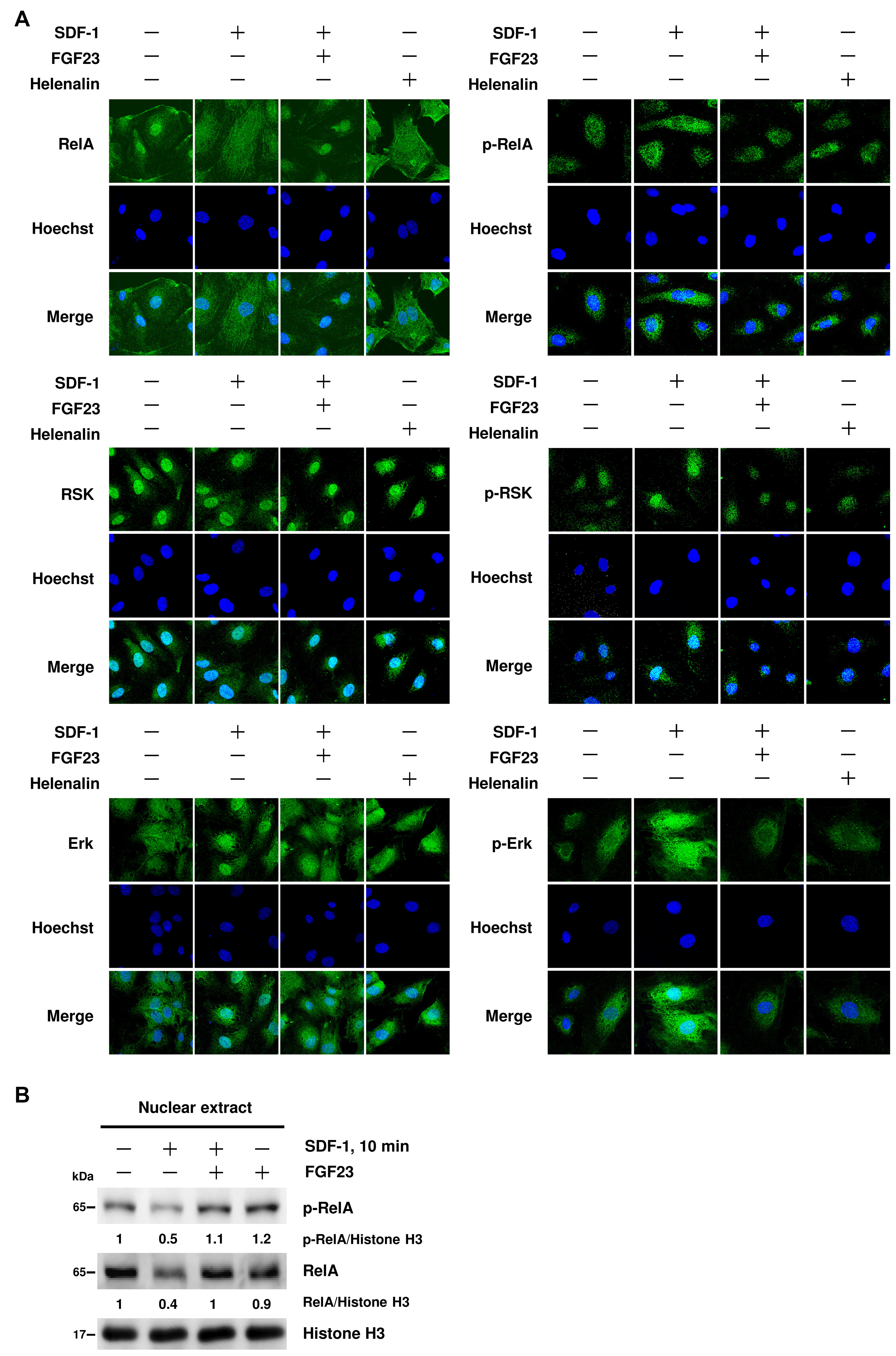

Supplement: Supplementary file 6 — Supplementary Fig. 5-1 [file 41419_2021_3693_MOESM6_ESM.tif]

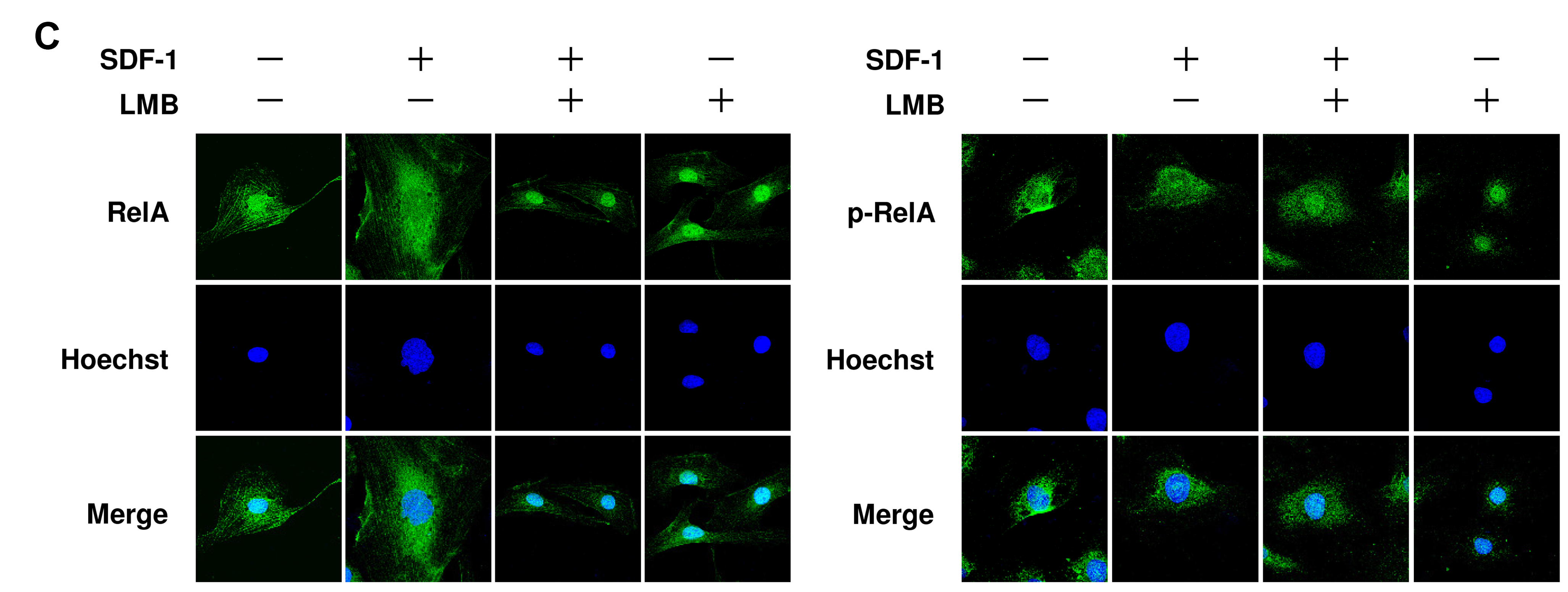

Supplement: Supplementary file 7 — Supplementary Fig. 5-2 [file 41419_2021_3693_MOESM7_ESM.tif]

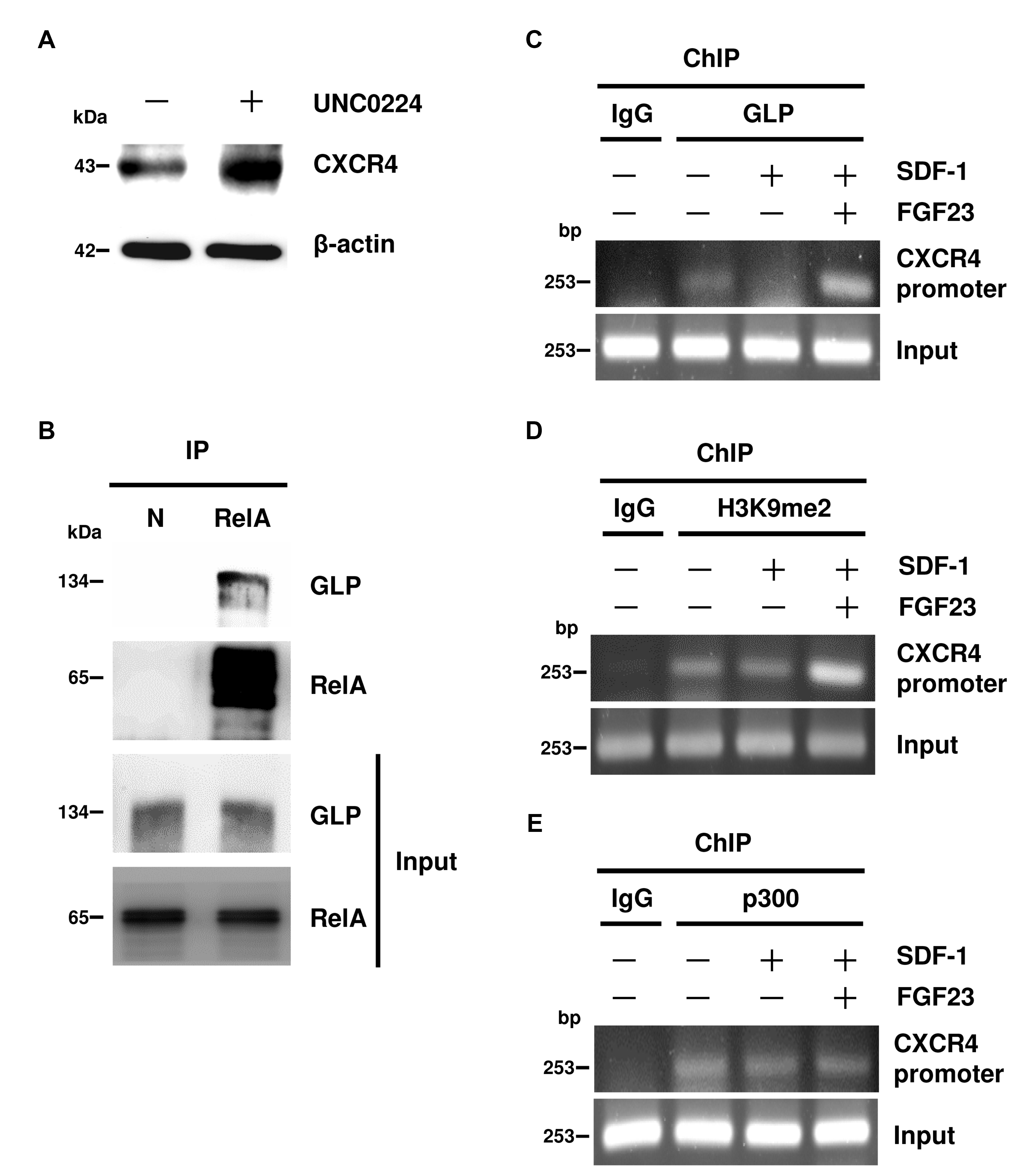

Supplement: Supplementary file 8 — Supplementary Fig. 6 [file 41419_2021_3693_MOESM8_ESM.tif]

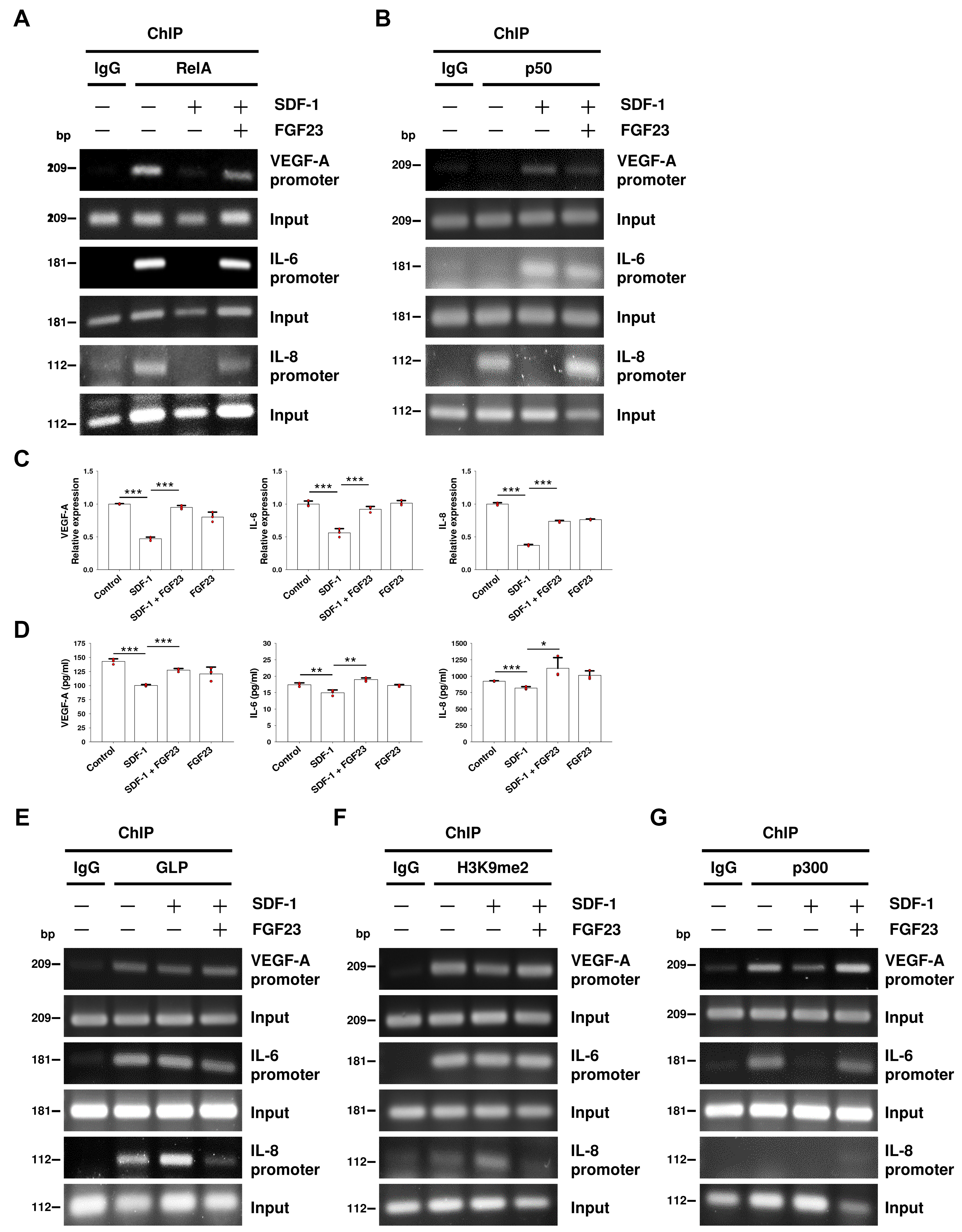

Supplement: Supplementary file 9 — Supplementary Fig. 7 [file 41419_2021_3693_MOESM9_ESM.tif]

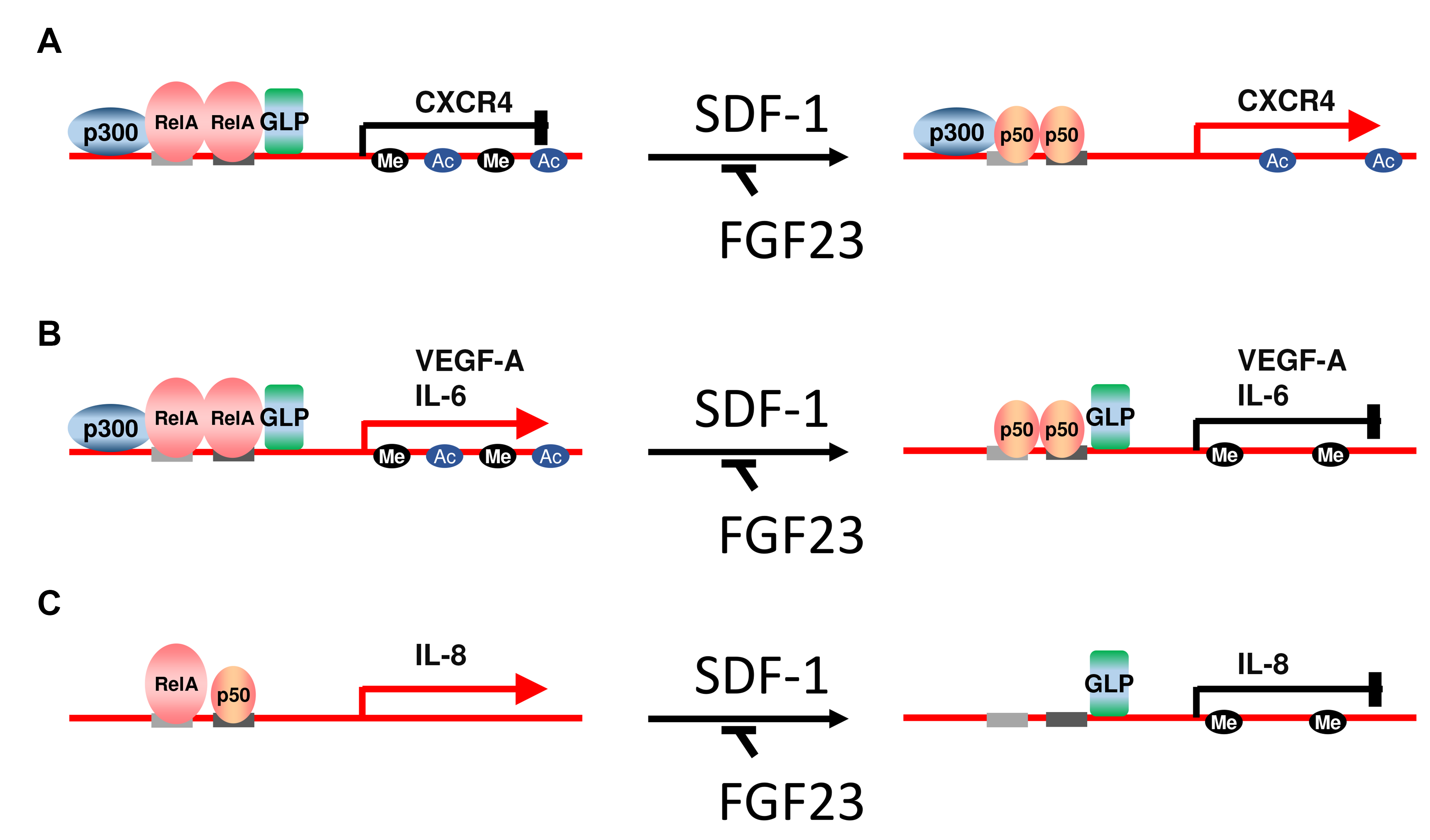

Supplement: Supplementary file 10 — Supplementary Fig. 8 [file 41419_2021_3693_MOESM10_ESM.tif]

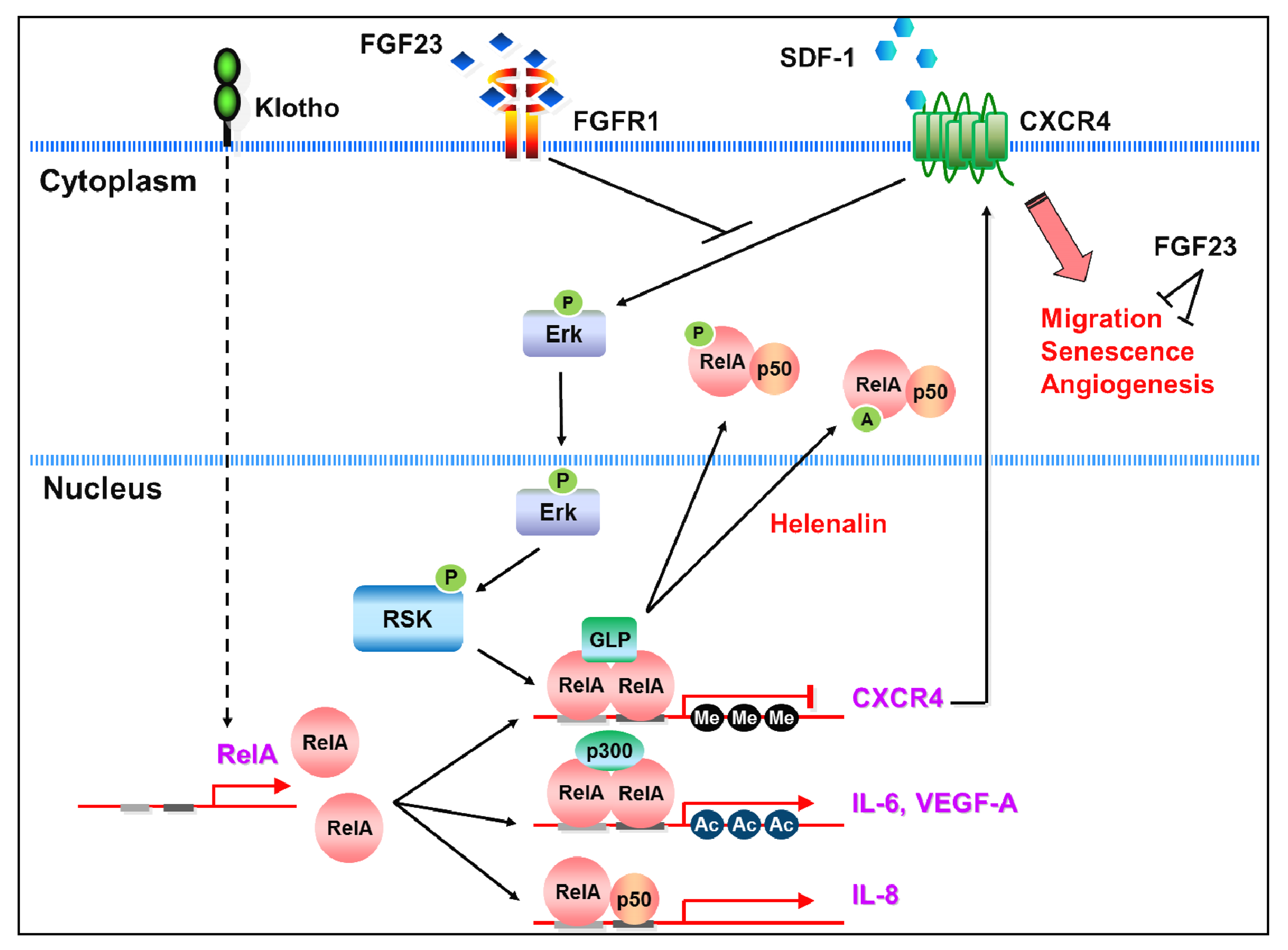

Supplement: Supplementary file 11 — Supplementary Fig. 9 [file 41419_2021_3693_MOESM11_ESM.tif]
